# Phytosulfokine downregulates defense‐related WRKY transcription factors and attenuates pathogen‐associated molecular pattern‐triggered immunity

**DOI:** 10.1111/tpj.17115

**Published:** 2024-12-11

**Authors:** Dian Liu, Joanna Jelenska, Jessica M. Morgan, Jean T. Greenberg

**Affiliations:** ^1^ Biochemistry and Molecular Biophysics The University of Chicago Chicago Illinois 60637 USA; ^2^ Department of Molecular Genetics and Cell Biology The University of Chicago Chicago Illinois 60637 USA; ^3^ Biophysical Sciences The University of Chicago Chicago Illinois 60637 USA

**Keywords:** *Arabidopsis thaliana* (L.) *Heynh.*, phytosulfokine, RNA‐seq, plant defense, WRKY, flg22, PAMP

## Abstract

Phytosulfokine (PSK) is a plant growth‐promoting peptide hormone that is perceived by its cell surface receptors PSKR1 and PSKR2 in Arabidopsis. Plants lacking the PSK receptors show phenotypes consistent with PSK signaling repressing some plant defenses. To gain further insight into the PSK signaling mechanism, comprehensive transcriptional profiling of Arabidopsis treated with PSK was performed, and the effects of PSK treatment on plant defense readouts were monitored. Our study indicates that PSK's major effect is to downregulate defense‐related genes; it has a more modest effect on the induction of growth‐related genes. WRKY transcription factors (TFs) emerged as key regulators of PSK‐responsive genes, sharing commonality with a pathogen‐associated molecular pattern (PAMP) responses, flagellin 22 (flg22), but exhibiting opposite regulatory directions. These PSK‐induced transcriptional changes were accompanied by biochemical and physiological changes that reduced PAMP responses, notably mitogen‐activated protein kinase (MPK) phosphorylation (previously implicated in WRKY activation) and the cell wall modification of callose deposition. Comparison with previous studies using other growth stimuli (the sulfated plant peptide containing sulfated tyrosine [PSY] and *Pseudomonas simiae* strain WCS417) also reveals WRKY TFs' overrepresentations in these pathways, suggesting a possible shared mechanism involving WRKY TFs for plant growth–defense trade‐off.

## Significance Statement

Our study reveals that phytosulfokine (PSK) promotes plant growth by suppressing defense mechanisms, highlighting a key link between growth regulation and immune response. By demonstrating how PSK downregulates WRKY TFs and associated kinase activity, this research provides valuable insights into plant biology, offering potential strategies for improving crop yield and pathogen resistance.

## INTRODUCTION

Plants have signaling systems that control their development and help them adapt to changing environments. In many cases, adaptation is mediated by non‐proteinaceous phytohormones like auxins and gibberellins (Davière & Achard, 2013; Leyser, 2018). However, many secreted peptides also play important roles in plant signaling via cell surface receptors (Boller, 2005; Czyzewicz et al., 2013; Matsubayashi, 2014).

Phytosulfokine (PSK) is a disulfated pentapeptide Tyr(SO_3_H)‐Ile‐Tyr(SO_3_H)‐Thr‐Gln (Matsubayashi & Sakagami, 1996). It is synthesized from prepropeptides encoded by five precursor genes (PSK1‐5) (Lorbiecke & Sauter, 2002; Yang et al., 2001). The prepropeptides are sulfated by tyrosylprotein sulfotransferase (TPST), which is a membrane‐bound enzyme localized in the *trans‐*Golgi network and encoded by a single gene in Arabidopsis (Komori et al., 2009). This enzyme also sulfates other plant peptides such as plant peptide containing sulfated tyrosine (PSY) and root growth meristem factor (Kaufmann & Sauter, 2019). Sulfated PSK precursors subsequently undergo proteolytic cleavage in apoplast and become active pentapeptides (Srivastava et al., 2008). In Arabidopsis, PSK is perceived by its receptors (PSKR1 and PSKR2) (Kutschmar et al., 2009; Stührwohldt et al., 2011) that reside at the plasma membrane (Hartmann et al., 2014). PSKRs are receptor‐like kinases with an extracellular domain, a single transmembrane helix, and an intracellular kinase domain (Matsubayashi et al., 2002). So far, no functional differences were reported for the two receptors, but PSKR1 is more well studied (Hartmann et al., 2013). The extracellular domain of PSKR1 consists of a leucine‐rich repeat (LRR) region with 21 LRRs, and a 36‐amino‐acid island domain intercepts LRRs 17 and 18 (Matsubayashi et al., 2002). A 15‐amino‐acid fragment of the island domain (Glu503‐Lys517) was identified as a ligand binding site (Shinohara et al., 2007) and PSK binding causes allosteric activation of PSKR1 (Wang et al., 2015). PSKR1 is a calmodulin‐binding protein that can associate with Ca^2+^ *in planta* (Muleya et al., 2014). Ca^2+^ regulates both the kinase and guanylate cyclase activities of PSKR1 *in vitro* (Irving et al., 2018). To date, the kinase substrates and cGMP‐regulated proteins, TFs, and gene targets have not been identified (Sauter, 2015).

Previous studies have illustrated PSK's role in promoting root elongation and leaf growth via cell division and cell growth promotion effects (Kutschmar et al., 2009; Matsubayashi et al., 2006; Yu et al., 2016). PSK signaling has been inferred to play an important part in regulating plant defense response. PSKR1 loss‐of‐function mutant *pskr1* displays enhanced disease symptoms and pathogen growth after infection with *Pseudomonas syringae* pv. tomato DC3000, suggesting PSK signaling attenuates immune responses induced by this bacterial pathogen (Mosher et al., 2013). However, the *pskr1* mutant is also more susceptible to the necrotrophic fungal infection with *Alternaria brassicicola*, indicating PSK signaling increases immune response induced by a fungal pathogen (Mosher et al., 2013). A later study demonstrated that the *pskr1* mutant shows autoimmunity in response to growth‐promoting *P. fluorescens*. Transcriptional profiling supported a role for PSKR1 in suppressing defenses regulated by the signal molecule salicylic acid (Song et al., 2023). Nonetheless, the transcriptional readouts of PSK's effects on plants are still lacking, and the relationship between transcriptomic changes and plant defense responses has yet to be well characterized.

In this study, we conducted comprehensive transcriptional profiling of Arabidopsis responses to PSK and uncovered PSK's role in mediating the trade‐off between plant growth and defense. WRKY TFs were over‐represented among the TFs regulating PSK‐responsive genes. Furthermore, these WRKY TFs shared with those involved in the response to flagellin 22 (flg22), a pathogen‐associated molecular pattern (PAMP) derived from bacterial flagellin (Felix et al., 1999), but were regulated in opposite directions by PSK and flg22. PSK was able to attenuate an early step of flg22‐induced signaling, MAP kinase activation, and reduce the deposition of callose, which is a key output of flg22 signaling. Notably, other growth‐promoting stimuli, including another TPST‐sulfated peptide called PSY and a beneficial bacterial strain *P. simiae* WCS417, modulated WRKY TFs with distinctive regulatory patterns.

## RESULTS

### PSK downregulates defense‐associated genes

To eliminate endogenous PSK signaling, we used synthetic peptide to treat *tpst* plants that cannot produce the native active sulfated PSK for transcriptional profiling. We reasoned that use of WT plants might obscure PSK's effects on the transcriptome due to signaling via endogenous PSK. We did transcriptional profiling of hydroponic *tpst* plants treated with synthetic PSK using RNA‐seq to capture gene expression readouts. Before profiling the plants, we validated that hydroponic *tpst* plants showed enhanced root growth in response to PSK (Figure S1). Next, we grew *tpst* plants for 11 days in hydroponic conditions and treated plants with 10 nM PSK for 30 min, 2 h, and 5 h to gain early responses to PSK; we also utilized *tpst* plants treated with PSK for 11 days to capture late responses to PSK. The roots and shoots were profiled separately to discern possible tissue‐specific responses to PSK at different time points. We performed additional RNA‐seq profiling of 7‐day‐old *tpst* plants treated with 100 nM or 1 μM PSK for 5 h and profiled the whole seedlings to investigate potential dose effects of PSK. These diverse experimental settings facilitated a comprehensive understanding of the PSK signaling pathway, allowing us to identify common genes and functional pathways responsive to PSK irrespective of plant age, tissue type, PSK concentration, or duration of treatment (Table S1).

**Figure 1:**
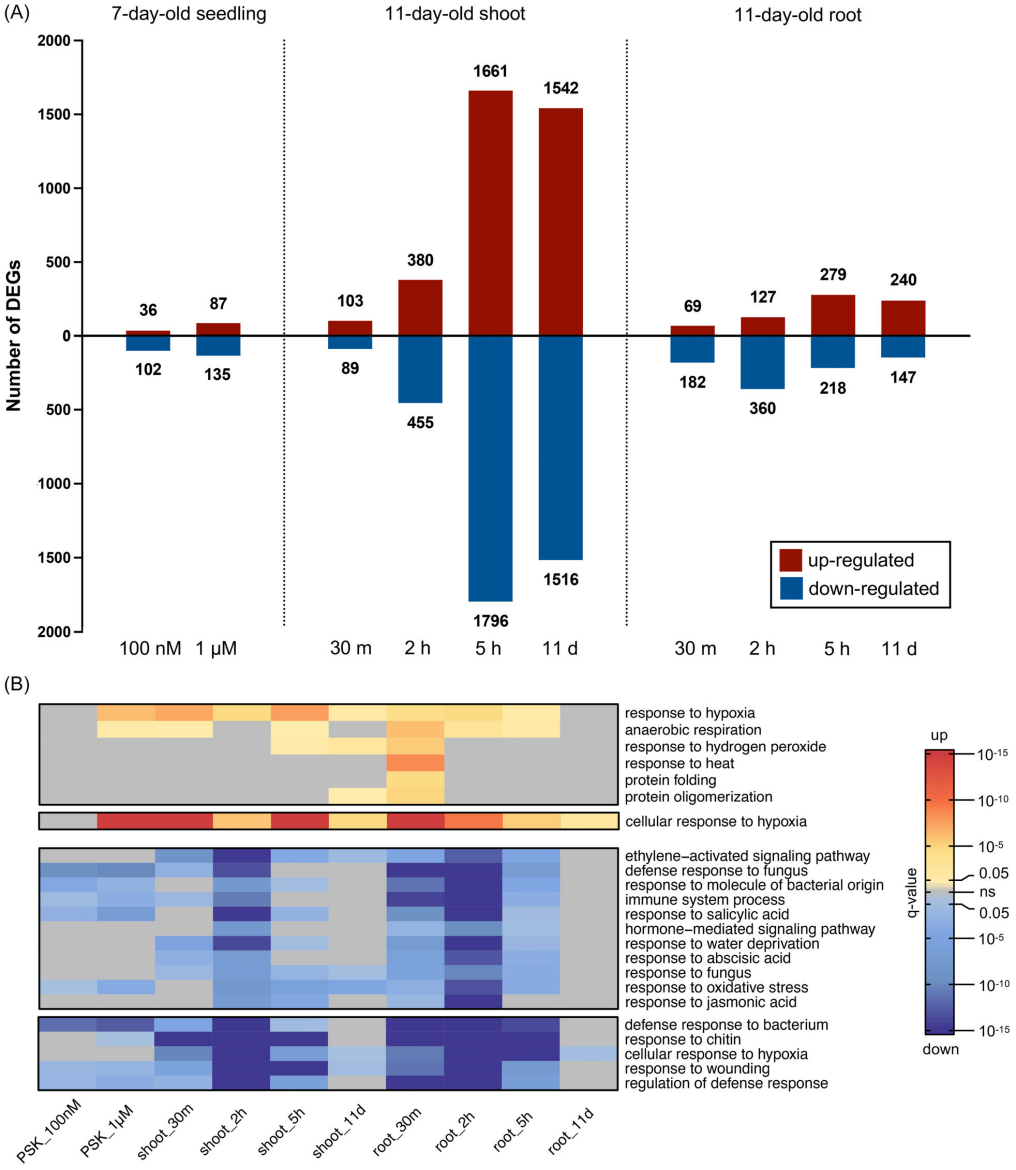
PSK effects on plant transcriptomics. (A) Number of DEGs after PSK treatments. Differential expression analysis was conducted by comparing PSK‐treated *tpst* plants with untreated or mock‐treated *tpst* plants. Red indicates upregulated genes, while blue indicates downregulated genes, using the cut‐off of ≥2‐fold change and adjusted *P*‐value <0.05. (B) Heatmap of most representative and common enriched GO_BP functional terms after PSK treatments (Cluster 3 from Figure S2A, Clusters 3 and 4 from Figure S2B, and the dendrograms were omitted). Red denotes terms enriched from upregulated DEGs, while blue denotes terms enriched from downregulated DEGs. The intensity of color reflects their significance measured by *q*‐value, utilizing the cut‐off of *q*‐value <0.05. Gray represents data points outside the cut‐off criteria.

Differential expression analysis was conducted by comparing WT plants and treated *tpst* plants with untreated or mock‐treated *tpst* plants with the cut‐off of ≥2‐fold change and adjusted *P*‐value <0.05 (Data S1). Figure 1A shows that longer exposure to PSK induced up to 18‐fold more differentially expressed genes (DEGs) compared to shorter treatment (e.g., 5 h vs. 30 m, shoot). Additionally, shoots exhibited up to 7.9‐fold more DEGs compared to roots at the same time points (e.g., 11 days, shoot vs. root, Figure 1A). When profiling whole seedlings, a higher concentration of PSK resulted in 61% more DEGs (seedling, 1 μM vs. 100 nM), indicating a dose‐dependent effect of PSK. However, the magnitude of transcriptional changes were smaller compared to the results obtained from profiling roots and shoots separately with plants at a later stage (e.g., 5 h, seedling vs. shoot/root, Figure 1A).

Functional enrichment analysis of DEGs in each PSK treatment and growth condition revealed numerous defense‐related gene ontology biological process (GO_BP) terms such as “defense response to bacterium,” “regulation of defense response,” and “immune system process,” along with important defense‐related hormone pathways such as “response to salicylic acid” and “response to jasmonic acid” were enriched from PSK downregulated DEGs. These defense‐associated functional terms were widely shared among various PSK treatment conditions (Figure 1B; Figure S2B). Many important genes related to plant defense, such as *FOX1*, *FOX5*, *MLO12*, *PCR8*, *IOS1*, *WRKY22*, *WRKY 72*, and *PME41*, were significantly downregulated by PSK (≥2‐fold change and adjusted *P*‐value <0.05; Figure S3).

Surprisingly, growth‐related GO_BP terms like “plant epidermis development” and “thalianol metabolic process” were only enriched from PSK‐upregulated DEGs in seedling samples with a limited number of DEGs (Figure S2A; Data S2). The thalianin pathway is associated with plant growth, and the overexpression of thalianol synthase *THAS* leads to longer roots (Field & Osbourn, 2008; Kaufmann et al., 2021). The core genes in thalianin pathway were upregulated by 100 nM and 1 μM PSK when profiled as whole seedling (Figure S3). However, these functional terms were not enriched from separately profiled root and shoot samples from plants at a later stage (Figure 1B; Figure S2A). Instead, hypoxia response‐related GO_BP terms like “response to hypoxia” and “anaerobic respiration” enriched from PSK‐upregulated DEGs were more commonly shared among different PSK treatment conditions, suggesting PSK has a wide upregulation effect on the expression of hypoxia‐related genes (Figure 1B; Figure S2A).

Taken together, the suppression effect of PSK on defense‐related genes is more prominent and consistent compared to its activation effect on growth‐related genes, both in terms of the number of DEGs and GO_BP terms enriched (Figure S2; Data S2 and S3).

### PSK‐mediated suppression of gene expression is highly associated with WRKY TFs

To identify the TFs that are likely to regulate PSK‐responsive genes, we conducted TF enrichment analysis of DEGs affected by PSK. WRKY TFs account for a significant portion of enriched TFs from PSK‐downregulated DEGs, constituting 88.5–100% of all enriched TFs in seedling samples, 5.6–34.1% in shoot samples, and 25.5–81.3% in root samples (Table 1; Data S4 and S5). WRKY TFs are one of the largest TFs families in plant and have 75 members in Arabidopsis (Chen et al., 2017; Rushton et al., 2010). WRKY TFs are characterized by the signature ‘WRKYGQK’ motif at N‐termini and a zinc finger motif at C‐termini in their DNA‐binding domains (Chen et al., 2017; Rushton et al., 2010). WRKY TFs play important roles in regulating plant response to both biotic and abiotic stress and are essential players in plant defense response (Javed & Gao, 2023; Wani et al., 2021).

**Table 1:** Number of enriched TFs, WRKY TFs, and percentage of WRKY TFs among all TFs from up‐ or downregulated DEGs with PSK, PSY, flg22, and WCS417 treatments

Treatment	TF enriched from upregulated DEGs				TF enriched from downregulated DEGs			
	All TF	WRKY	Percent	*P*‐value*	All TF	WRKY	Percent	*P*‐value*
PSK 100 nM seedling	15	0	0.0	1	29	29	100.0	<2.2e‐16
PSK 1 μM seedling	20	0	0.0	1	26	23	88.5	<2.2e‐16
PSK shoot 30 m	28	0	0.0	0.63	44	15	34.1	4.4e‐11
PSK shoot 2 h	52	0	0.0	0.17	80	26	32.5	<2.2e‐16
PSK shoot 5 h	70	0	0.0	0.12	54	3	5.6	0.49
PSK shoot 11 days	40	0	0.0	0.41	29	4	13.8	0.031
PSK root 30 m	45	0	0.0	0.26	32	26	81.3	<2.2e‐16
PSK root 2 h	12	0	0.0	1	51	13	25.5	5.6e‐08
PSK root 5 h	55	0	0.0	0.17	32	17	53.1	<2.2e‐16
PSK root 11 days	51	2	4.0	1	9	7	77.8	5.7e‐09
PSY shoot	13	5	38.5	0.00011	76	8	10.5	0.012
PSY root	0	0	0.0	1	45	27	60.0	<2.2e‐16
flg22 seedling	77	41	53.2	<2.2e‐16	123	1	0.1	0.058
WCS417 shoot	1	0	0.0	1	8	0	0.0	1
WCS417 root	39	21	53.8	<2.2e‐16	12	0	0.0	1

* The *P*‐value represents Fisher's exact test for the overrepresentation of enriched WRKY TFs among all TFs compared to the genome background (Data S4 and S5).

Notably, no WRKY TFs were enriched from PSK‐upregulated DEGs, except for two WRKY TFs identified in root samples exposed to PSK for 11 days (Table 1). The signature binding motifs of WRKY TF target genes, W‐boxes (TTGACT/TTGACA), were predominantly overrepresented in the promoter regions of PSK‐downregulated DEGs, but not in PSK‐upregulated DEGs (Table S2). This aligns with the findings of the TF enrichment analysis.

Taken together, we infer that WRKY TFs play significant regulatory roles in PSK‐downregulated DEGs, while not in PSK‐upregulated DEGs. Additionally, the *TPST* gene, all *PSK* precursors genes (*PSK1‐5*), and *PSK* receptors genes (*PSKR1‐2*) possess at least one of the two W‐box signature binding motifs in their promoter regions (Table S3). There is experimental evidence that *PSK3*, *4*, and *5* are targeted by WRKY40, and *PSKR1* is targeted by WRKY33 (Birkenbihl et al., 2017), indicating WRKY TFs may directly regulate genes involved in the PSK pathway. Consistent with this, we observed that the expression levels of *PSK1*, *PSK4*, and *PSKR1* were downregulated by PSK by approximately 40% across various treatment conditions (Figure S3; Data S1).

The Arabidopsis transcriptional regulatory map (ATRM) provides a high‐quality ATRM transcriptional regulatory map that covers 388 TFs with strong supporting evidence from published references (Tian et al., 2020). Degree centrality measures the number of neighbors each node has, and closeness centrality measures the impact of a certain node in biological networks (Evans & Chen, 2022). By integrating ATRM's manually curated high‐confidence dataset, we were able to assess the degree centrality and closeness centrality of WRKY TFs among these annotated TFs (Figure S4A). Most WRKY TFs within this network exhibited relatively high levels of degree centrality, suggesting they are among the TFs with the most interconnected nodes. Additionally, they displayed very high levels of closeness centrality, indicating their roles as rapid communicators within the network. This observation aligns with our findings from the TF enrichment analysis, highlighting the influential impact of WRKY TFs in the regulatory network.

We integrated regulatory effects from the ATRM dataset and constructed a regulatory network for WRKY TFs (Figure S4B). Many important plant defense genes in this network, such as *PR1*, *FRK1*, *AOS*, *LOX2*, *LOX3*, *PDF1.2*, *BGL2*, *LURP1*, *ORA59*, *ERF1*, *ERF4*, *HSFB2A*, and *HSFB2B*, are regulated by WRKY TFs, and they were downregulated by PSK (Figure S5), indicating WRKY TFs' role in PSK‐mediated defense gene suppression.

### PSK and flg22 target a similar set of WRKY TFs and genes but with opposite regulatory effects on their expression

**Figure 2:**
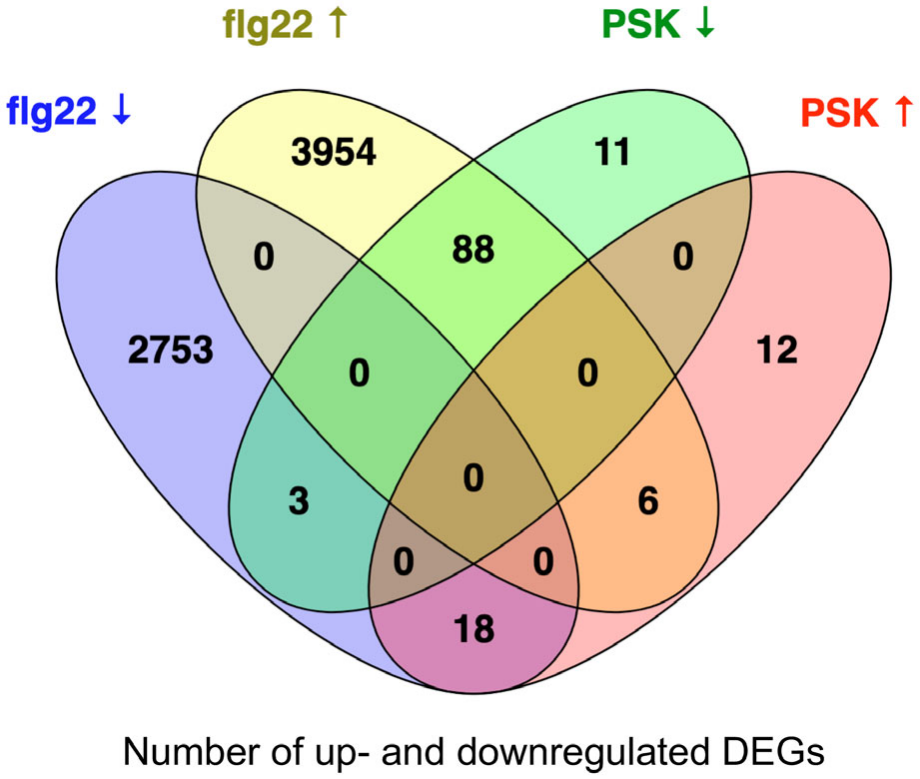
Venn diagram of the number of up‐ or downregulated DEGs induced by PSK and flg22 (Table S4; Data S6).

flg22 induces a broad spectrum of plant defense genes and physiological responses (Denoux et al., 2008), whereas our transcriptomic analysis revealed that PSK elicits the opposite effects. A comparison of PSK‐induced DEGs with previously found flg22‐induced DEGs (Birkenbihl et al., 2017) revealed intriguing patterns (Table 2). In our seedling samples, 86.3% (88 of 102) of downregulated DEGs induced by 100 nM PSK were upregulated by flg22, and 50.0% (18 of 36) of PSK upregulated DEGs induced by PSK were downregulated by flg22 (Figure 2; Table 2; Data S6). A significant proportion of PSK‐induced DEGs—76.8% (106 of 136)—overlapped with DEGs induced by flg22 but with opposite directions (Table 2). Across various PSK treatments, ranging from 59.5 to 76.8% of DEGs were oppositely regulated by PSK and flg22 in seedling samples, 18.1–48.0% in shoot samples, and 39.5–63.8% in root samples (Figure S6). Irrespective of PSK treatment methods or plant tissue, a considerable number of DEGs consistently exhibited opposite regulatory patterns in response to flg22.

**Table 2:** Summary of number of DEGs in each category (see Figure 2) and their percentages among DEGs induced by PSK. Of a total of 138 PSK‐modulated DEGs, 36 were upregulated and 102 were downregulated by PSK (Data S6)

Category	Number*	Total†	Percentage
flg22 ↑ PSK ↓	88	102	86.3
flg22 ↓ PSK ↓	3	102	2.9
flg22 ‐ PSK ↓	11	102	10.8
flg22 ↓ PSK ↑	18	36	50.0
flg22 ↑ PSK ↑	6	36	16.7
flg22 ‐ PSK ↑	12	36	33.3
Opposite to flg22	106	138	76.8
Same as flg22	9	138	6.5
Did not respond to flg22	23	138	16.7

* The number of genes with category pattern.

^†^ The total number of PSK‐regulated genes.

**Figure 3:**
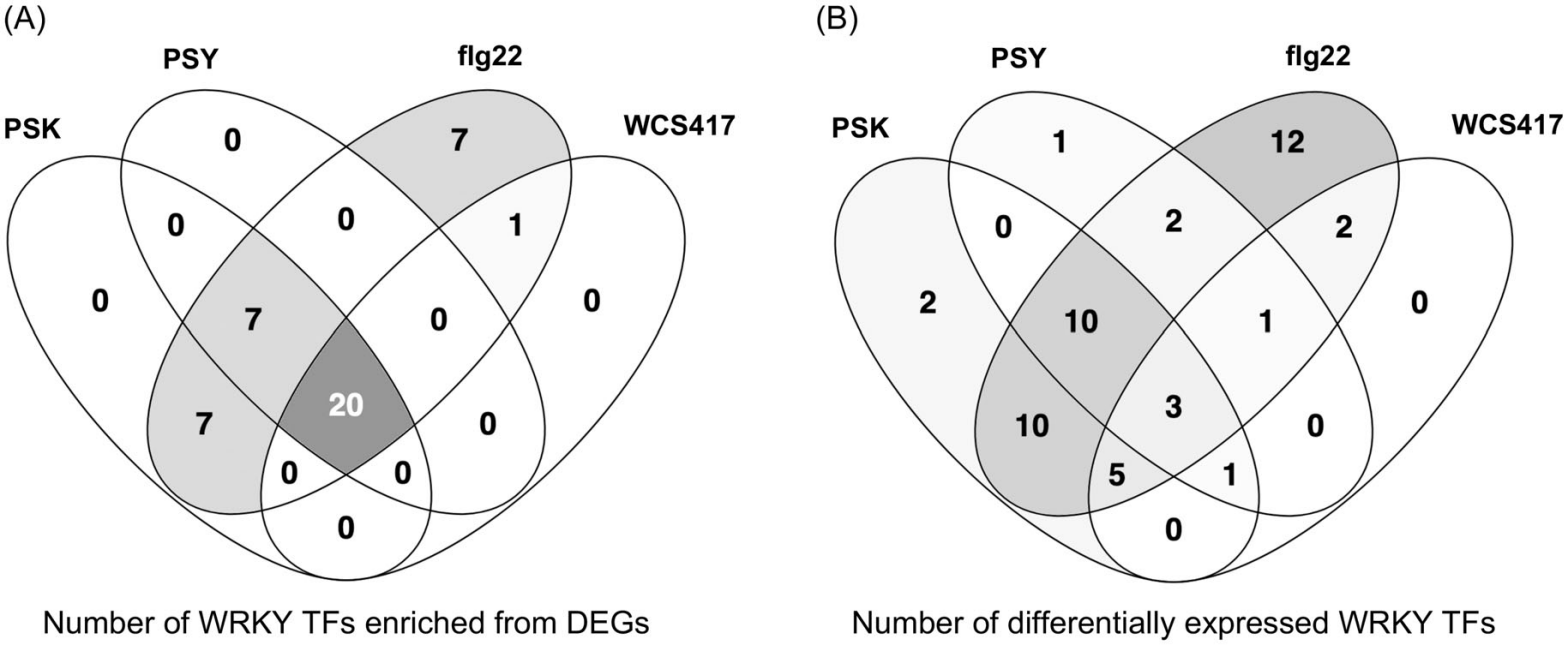
Enriched WRKY TFs and differentially expressed WRKY TFs comparisons among stimuli. (A) Venn diagram of the number of WRKY TFs enriched from DEGs in PSK, PSY, flg22, and WCS417 treatments (Data S7). (B) Venn diagram of the number of differentially expressed WRKY TFs in PSK, PSY, flg22, and WCS417 treatment conditions (Data S7).

**Figure 4:**
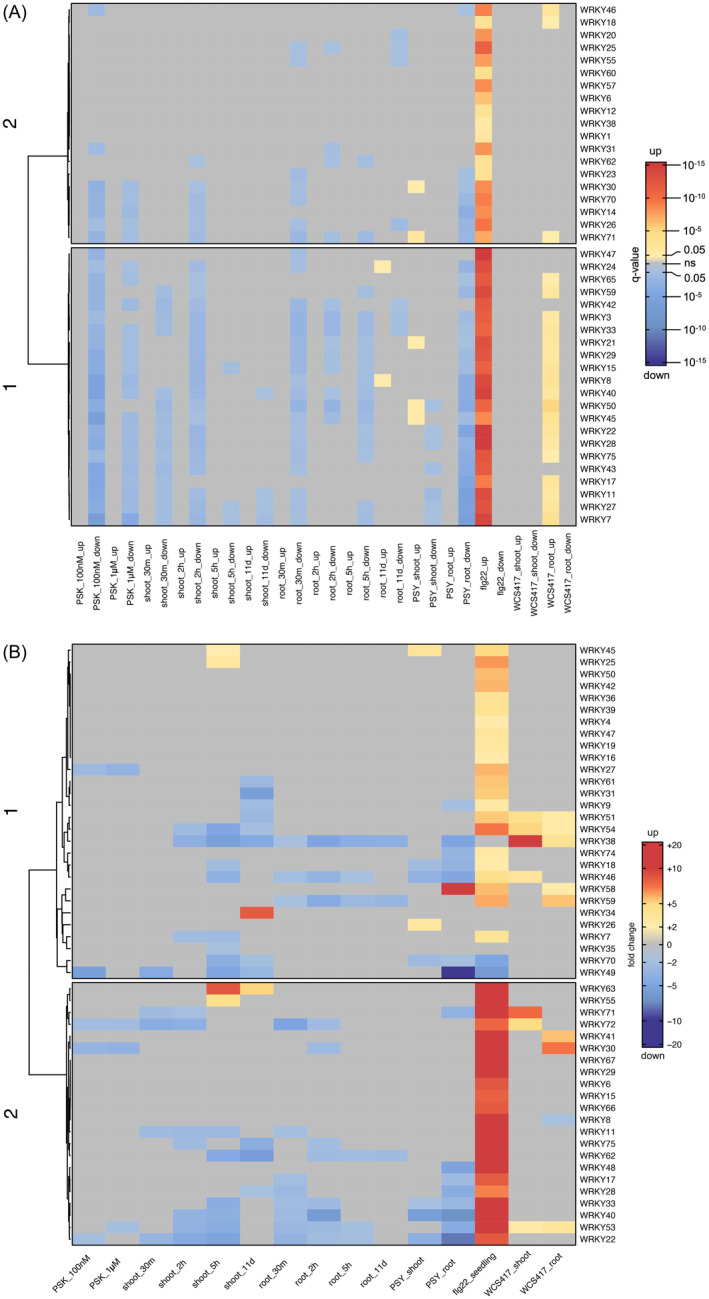
Heatmaps of WRKY TFs enrichment and expression among multiple stimuli. (A) Heatmap of enriched WRKY TFs from PSK, PSY, flg22, and WCS417 up‐ and downregulated DEGs. The dendrogram on the side of the heatmap illustrates the hierarchical clustering of rows using k‐means clustering with numbers on the branches denoting the distance measures. Red denotes enrichment from upregulated DEGs, while blue denotes enrichment from downregulated DEGs. The intensity of color reflects their significance measured by *q*‐value, utilizing the cut‐off of *q*‐value <0.05. Gray represents data points outside the cut‐off criteria. (B) Heatmap of differentially expressed WRKY TFs with PSK, PSY, flg22, and WCS417 treatments. The dendrogram on the side of the heatmap illustrates the hierarchical clustering of rows using k‐means clustering with numbers on the branches denoting the distance measures. Red indicates upregulation, while blue indicates downregulation. The intensity of color reflects their fold changes, utilizing the cut‐off of ≥2‐fold change and adjusted *P*‐value <0.05. Gray represents data points outside the cut‐off criteria.

We further compared the differentially expressed *WRKY* TF genes and WRKY TFs enriched from DEGs that may target their downstream genes in PSK and flg22 cases (Figure 3; Table 3; Data S7). Among the 31 differentially expressed *WRKY* TF genes in PSK‐treated plants and 45 differentially expressed *WRKY* TF genes in flg22‐treated plants, 28 were found to be the same WRKY TFs. Notably, 22 of these 28 shared WRKY TFs were regulated by PSK and flg22 in opposite directions (Figure 4B; Data S8). All the 34 WRKY TFs enriched from PSK‐induced DEGs were included within the 42 WRKY TFs enriched from flg22‐induced DEGs, and these shared WRKY TFs were enriched from PSK‐downregulated DEGs and flg22‐upregulated genes (Figure 4A; Data S8).

**Table 3:** Summary of the total number of enriched WRKY TFs from DEGs in PSK, PSY, flg22, and WCS417 treatments and their ratio over all WRKY TFs in the genome (Data S7)

Treatment	Enriched WRKY TFs in all enriched TFs				Differentially expressed WRKY TFs in all DEGs			
	Number	Ratio	Background*	*P*‐value^†^	Number	Ratio	Background*	*P*‐value^†^
PSK	34	34/72	279/1717	2.9e‐10	31	31/72	5196/32 833	3.6e‐08
PSY	27	27/72	111/1717	1.5e‐15	18	18/72	1382/32 833	7.4e‐10
flg22	42	42/72	196/1717	<2.2e‐16	45	45/72	6823/32 833	1.9e‐14
WCS417	21	21/72	60/1717	1.3e‐15	12	12/72	2304/32 833	0.0041

* The background shows the ratio of the total number of enriched TFs over all TFs in the genome.

^†^ The *P*‐value represents Fisher's exact test for the overrepresentation of enriched WRKY TFs among all WRKY TFs compared to all enriched TFs among the genome background.

Overall, PSK primarily downregulated *WRKY* TF genes, while flg22 upregulated this similar set of *WRKY* TF genes. Moreover, from the TF enrichment analysis, the common WRKY TFs were enriched from PSK‐downregulated DEGs, while from flg22‐upregulated DEGs. This is further supported by the fact that WRKY TFs were only overrepresented in flg22‐upregulated genes, but not in the downregulated genes (Table 1).

**Figure 5:**
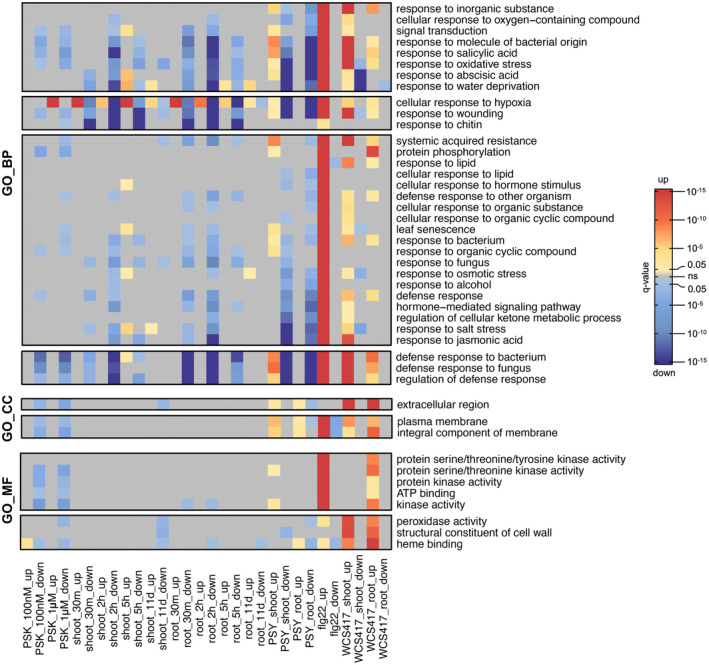
Heatmap of most representative and common enriched GO_BP, GO_CC, and GO_MF functional terms with PSK, PSY, flg22, and WCS417 treatments (clusters 1, 2, 4, and 5 from Figure S7A, clusters 2 and 3 from Figure S7B, clusters 2 and 3 from Figure S7C, and the dendrograms were omitted). Red denotes functional terms enriched from upregulated DEGs, while blue denotes functional terms enriched from downregulated DEGs. The intensity of color reflects their significance measured by *q*‐value, utilizing the cut‐off of *q*‐value <0.05. Gray represents data points outside the cut‐off criteria.

From functional enrichment analysis, the majority of shared GO_BP terms between PSK and flg22 were related to plant defense. However, PSK downregulated genes that are associated with plant defense, whereas flg22 upregulated genes within these functional terms (Figure 5; Figure S7A).

### PSK attenuates some responses to flg22

The common WRKY TFs shared by PSK and flg22 and their opposite regulatory effects on plant defense‐related genes suggest a potential counteractive role of PSK against the impact of flg22 on plant defense responses. Therefore, we tested which signaling steps upstream of gene expression may be targeted by PSK.

**Figure 6:**
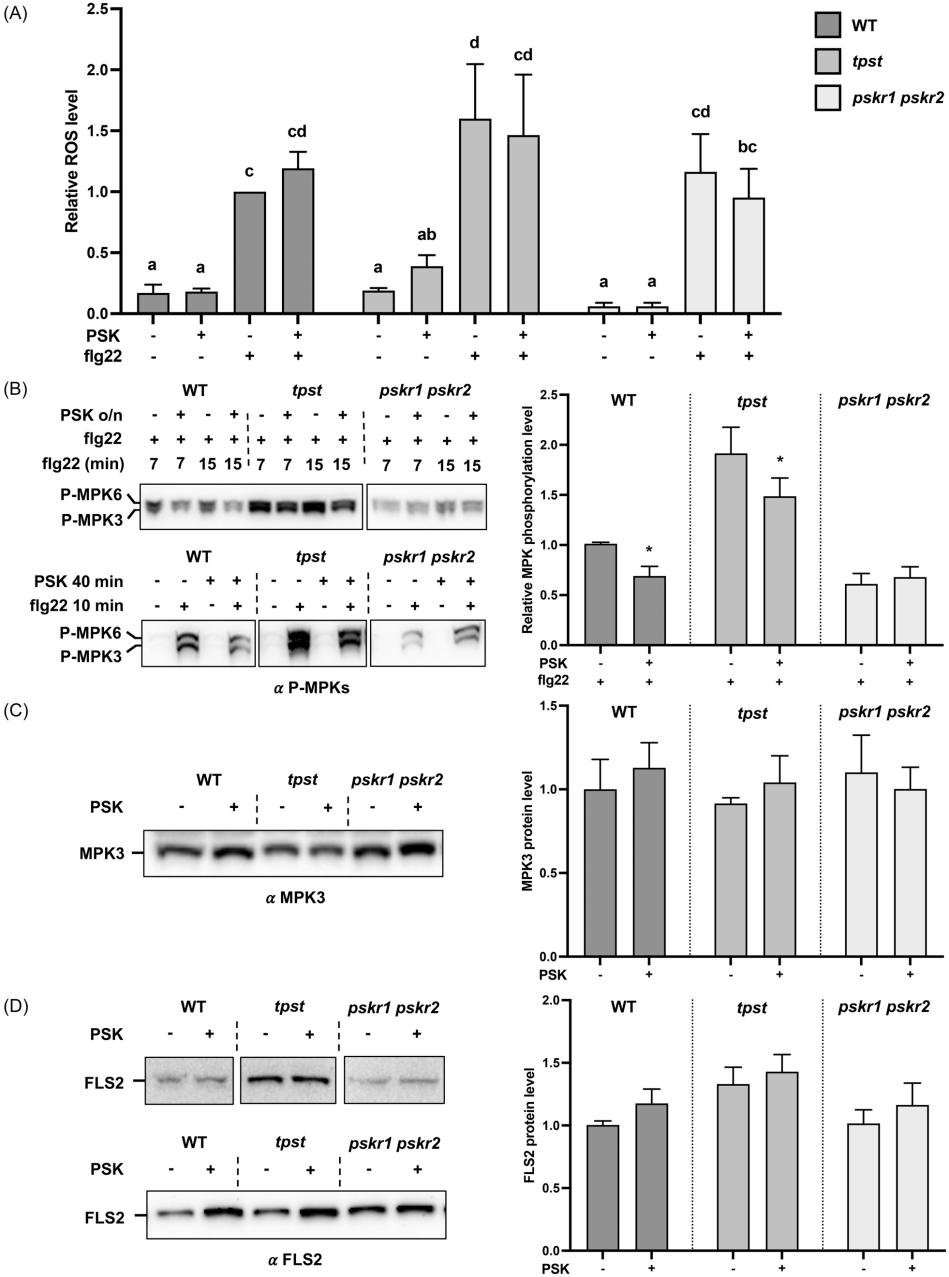
Effect of PSK on several flg22‐induced outputs. (A) PSK pretreatment does not affect flg22‐induced ROS. *tpst* plants have higher ROS response than WT (Figure S8A). The mean values of relative ROS accumulation from several experiments are shown (see “Methods” section). Values are relative to WT, which was set to “1.” Different letters indicate significant differences in one‐way ANOVA analysis (*P*‐value <0.05, Fisher's test). (B) Effect of PSK on flg22‐induced MPK phosphorylation. Immunoblots show phospho‐MPK3 and ‐MPK6 in seedlings pretreated overnight with PSK and subsequently induced by flg22 for 7 and 15 min (upper blot, 200 nM peptides) and in seedlings treated for 40 min with PSK and 10 min with flg22 (lower blot, 1 μM peptides). Mock treatment was growth medium with water added instead of peptide(s). In both panels, samples are from two gels run, blotted and exposed together. PSK pretreatment reduced flg22‐induced MPK phosphorylation. *tpst* plants showed higher MPK phosphorylation than WT and *pskr1 pskr2* (Figure S8B). MPK3 and 6 were not phosphorylated without flg22 induction. Graph shows mean MPK phosphorylation from several experiments relative to WT with flg22 treatment only. Asterisk indicates ANOVA/Fisher (for pairs) *P*‐value <0.05, *n* = 5 (3 for *tpst*). (C) PSK pretreatment does not affect MPK3 protein level. Left panel shows immunoblot with MPK3 antibody of plant samples treated overnight with 1 μM PSK. Graph, mean MPK3 protein level relative to WT without PSK treatment. *P*‐value >0.05, two‐way ANOVA/Fisher or *t*‐test, *n* = 4 (3 for *tpst*). See also Figure S8C. (D) PSK treatment does not significantly change FLS2 protein level. *tpst* plants have higher FLS2 protein level than WT and *pskr1 pskr2* (Figure S8D). Left panel shows immunoblots with FLS2 antibody of plant samples treated overnight with 200 nM flg22. All samples are from the same gel/blot. In some experiments, there was some increased FLS2 in response to PSK in WT and *tpst* plants (compare upper and lower blots), but when all the data were combined on average, there was only a small increase. Graph, mean FLS2 protein level relative to WT without PSK treatment. *P*‐value >0.05, two‐way ANOVA/Fisher or *t*‐test, *n* = 14 (10 for *tpst*). FLS2 protein was analyzed in the same experiments as MPK phosphorylation and basal level.

One of the very early responses to flg22, the reactive oxygen species (ROS) burst (Felix et al., 1999; Tateda et al., 2014), was mostly unaffected by PSK (Figure 6A). Another early response to flg22, MAP kinase phosphorylation, is upstream of defense gene expression activation (Lassowskat et al., 2014; Wani et al., 2021). All genotypes tested (WT, *tpst*, and *pskr1 pskr2*) showed flg22‐induced phosphorylation of MAP kinases (Figure 6B), although the absolute induction was higher in *tpst* and lower in *pskr1 pskr2* relative to WT (Figure S8B). When pretreated with PSK, flg22‐induced phosphorylation was attenuated by approximately 30% (Figure 6B), while the basal level of MPK3 was unaffected (Figure 6C; Figure S8C). These effects on phosphorylation were receptor dependent (see *pskr1 pskr2* response in Figure 6B). MPK3/6 can phosphorylate several WRKY TFs including WRKY33 and WRKY18 (Wang et al., 2018, 2023). We found that PSK downregulates the expression of *WRKY18* and *WRKY33* while flg22 upregulates them, indicating opposite regulatory directions (Figure 4B). This connects MAP kinase phosphorylation and the signaling pathways mediated by WRKY TFs.

Suppression of PAMP responses may result from altered receptor levels, therefore we tested levels of the flg22 receptor FLS2 after PSK treatment. Surprisingly, we found that FLS2 was slightly more abundant in WT and *tpst* tissues treated with PSK (Figure 6D). The effect was sometimes more pronounced (Figure 6D lower blot), but when multiple experiments were quantified and combined, the effect was small. PSK can attenuate ubiquitination and degradation of its own receptor PSKR1 (Hu et al., 2023). It is plausible that PSK stabilizes other receptors such as FLS2 and prevents their endocytosis, which is crucial for FLS2 signaling (Mbengue et al., 2016; Salomon & Robatzek, 2006; Smith et al., 2014; Spallek et al., 2013). *tpst* plants were more sensitive to flg22 treatment, as they showed higher ROS burst and MPK phosphorylation, and had higher basal level of FLS2 protein than wild‐type plants (Figure S8).

**Figure 7:**
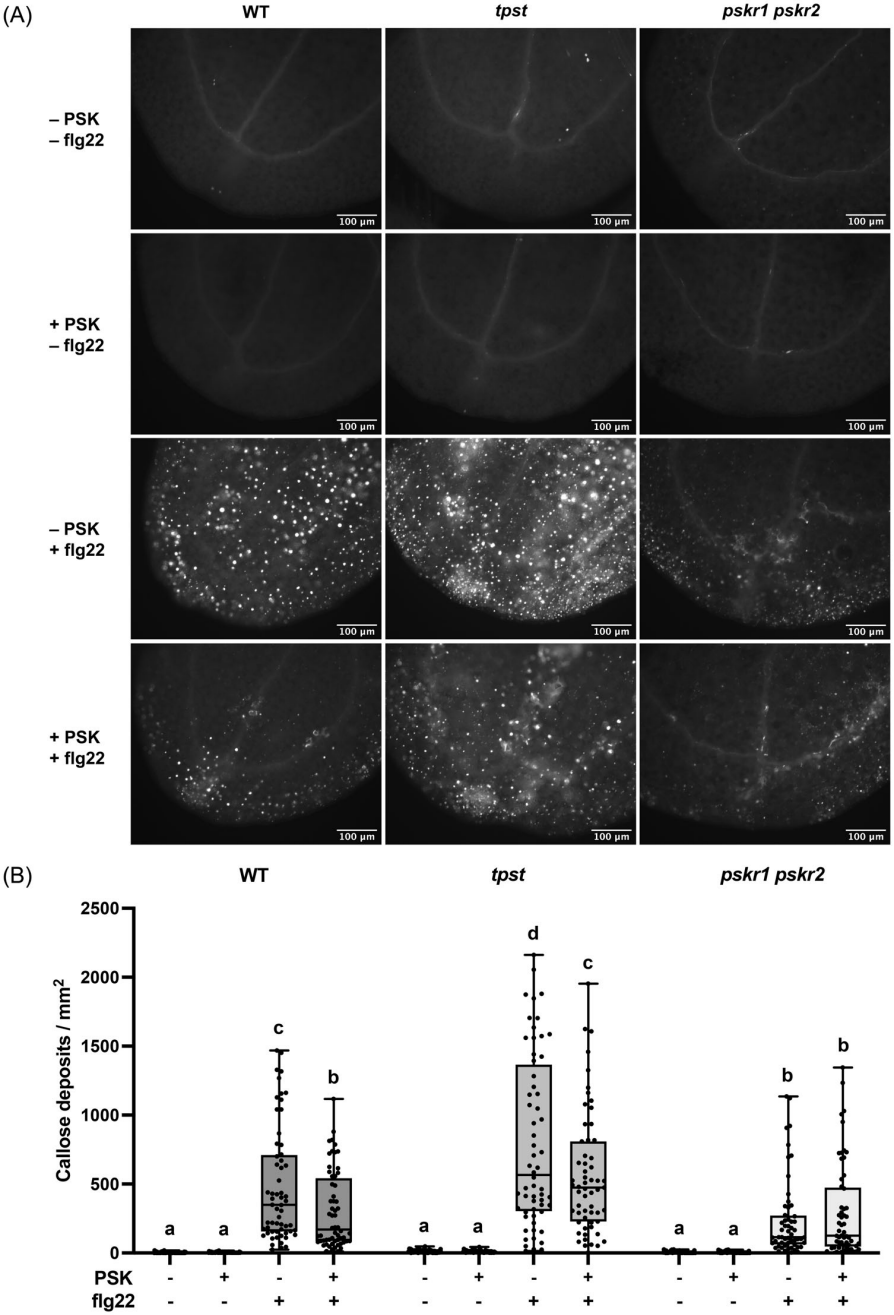
PSK reduces flg22‐induced callose deposition. (A) Callose deposition visualized by aniline blue staining and fluorescence microscopy in WT, *tpst*, and *pskr1 pskr2* plants treated with or without PSK and flg22. (B) Callose density quantified by the number of callose deposits per mm^2^ in WT, *tpst*, and *pskr1 pskr2* plants treated with or without PSK and flg22. Each group contains at least 52 cotyledons with high‐quality images from 30 seedlings, which were pooled for analysis. Different letters indicate significant differences in one‐way ANOVA analysis (*P*‐value <0.05, Tukey's test).

We further examined flg22‐induced deposition of callose, which is the polysaccharide that plants form in the cell wall and plasmodesmata regions in response to stimuli such as pathogens and stress (German et al., 2023). Prior to treating WT, *tpst*, and *pskr1 pskr2* seedlings with 1 μM flg22 or mock treatments of water for 24 h on day 8, we implemented a 24‐h pretreatment on day 7 with 100 nM PSK or water (for mock treatments). The density of flg22‐induced callose deposits was significantly reduced by PSK pretreatment in WT and *tpst* plants, but not in *pskr1 pskr2* plants, indicating a PSKR‐dependent suppression by PSK (Figure 7). The relative area of callose deposition also displayed this PSK‐ and PSKR‐dependent trend, while the average size of callose deposits remained similar across the three genotypes (Figure S9).

Taken together, PSK pretreatment reduces the number of flg22‐induced callose deposits only in the presence of PSKRs, with no significant effects on callose deposit sizes. A possible molecular explanation at the gene expression level for this effect is through regulation of the *ATL31* gene, which positively contributes to callose deposition (Maekawa et al., 2012). *ATL31* is targeted by WRKY18, 40, and 33 (Birkenbihl et al., 2017), and both the expression of *ATL31* and its upstream regulators *WRKY18*, *40*, and *33* were downregulated by PSK, but upregulated by flg22 (Figure 4B; Figure S3). WRKY18 and WRKY33 are phosphorylated by MPK3/6 (Wang et al., 2018, 2023), whereas the phosphorylation of MPK3/6 was reduced by PSK (Figure 6B). Our results are consistent with the report that *mpk3* mutant plants, which phenocopied the suppression of MPK3 phosphorylation, have reduced flg22‐induced callose deposition (Frei Dit Frey et al., 2014).

### PSK, PSY, flg22, and WCS417 responses share a similar set of WRKY TFs but exhibit different effects on plant defense

The interplay between the PSK and flg22 pathways prompted further investigation into whether these opposing effects stem from the regulatory influence of WRKY TFs on plant defense. We conducted an integrated analysis utilizing published RNA‐seq data from plants treated with other growth or immune‐modulating stimuli. This included PSY (Ogawa‐Ohnishi et al., 2022), another TPST‐sulfated growth‐promoting peptide, and *P. simiae* strain WCS417 (Desrut et al., 2020), a beneficial bacterium that promotes plant growth and primes plant immunity, in addition to the PAMP discussed above, flg22, to elucidate their effects on WRKY TFs and plant defense (Table S4). This comparison aimed to identify potential high‐level regulators shared across these different treatments.

At the gene expression level, most *WRKY* TF genes were downregulated by PSK and PSY, but upregulated by flg22 and WCS417 (Figure 4B). Furthermore, TF enrichment analysis revealed that the majority of WRKY TFs were exclusively enriched from downregulated DEGs of PSK and PSY treatments, and from upregulated DEGs of flg22 and WCS417 treatments, not vice versa (Figure 4A). The significant overlap observed between any two of the differentially expressed WRKY TFs from PSK, PSY, flg22, and WCS417 treatments, as well as WRKY TFs enriched from DEGs in each case, underscores the existence of a common regulatory network underlying these four distinct treatments, rather than a mere coincidental sharing of a similar set of WRKY TFs (Figure 3; Tables 3 and 4).

**Table 4:** Number of overlapping enriched WRKY TFs and differentially expressed WRKY TFs shared by PSK, PSY, flg22, and WCS417 treatments (Data S7)

Comparison	Enriched WRKY TFs in all enriched TFs				Differentially expressed WRKY TFs in all DEGs			
	Left*	Overlap*	Right*	*P*‐value^†^	Left*	Overlap*	Right*	*P*‐value^†^
PSK ∩ PSY	34	27	27	<2.2e‐16	31	14	18	8.4e‐05
PSK ∩ flg22	34	34	42	<2.2e‐16	31	28	45	1.1e‐06
PSK ∩ WCS417	34	20	21	1.2e‐09	31	9	12	0.0026
PSY ∩ flg22	27	27	42	<2.2e‐16	18	16	45	0.00076
PSY ∩ WCS417	27	20	21	3.8e‐13	18	5	12	0.039
flg22 ∩ WCS417	42	21	21	<2.2e‐16	45	11	12	0.0019

* Left and right indicate the number of WRKY TFs as the element of comparison on the left and the right, respectively. Overlap shows the number of common WRKY TFs.

^†^ The *P*‐value represents the hypergeometric probability of randomly selecting the desired number of WRKY TFs from all WRKY TFs in left or right category and having at least the desired number of overlapping across 100 000 simulations, given the hypothesis that they are independent.

WRKY TFs are a distinctive cluster of all enriched TFs from DEGs of PSK, PSY, flg22, and WCS417 treatments (Figure S10). The overrepresentation of WRKY TFs and the presence of the WRKY TFs' signature W‐box motif also conformed to the observed pattern of “same WRKY TFs but opposite directions” (Table 1; Table S2). Specifically, WRKY TFs associated with PSK and PSY treatments are predominantly overrepresented in downregulated DEGs, whereas they are primarily enriched from upregulated DEGs with flg22 and WCS417 treatments.

The overlapping GO_BP terms among these four treatments predominantly pertain to defense‐related responses, such as “defense response to bacterium,” “regulation of defense response,” and “response to molecule of bacterial origin,” as well as other defense‐related hormone or signaling pathways like “response to salicylic acid,” “response to jasmonic acid,” and “systemic acquired resistance” (Figure 5; Figure S7A). Collectively, these findings indicate that PSK and PSY suppress plant defense responses, while flg22 and WCS417 induce plant defense responses, thus corroborating our previous understanding of the distinct effects of these four treatments and pathways (Hu et al., 2018; Pieterse et al., 2021; Wu et al., 2014).

Notably, the transcriptomic changes induced by PSK treatment were more similar to those observed with PSY treatment, as indicated by fewer DEGs being regulated in opposite directions (Figure S6). Additionally, their effects on the expression levels of WRKY TFs and defense‐related GO_BP terms were more alike when compared to the effects of flg22 and WCS417 (Figures 4 and 5). However, the GO_BP terms such as “protein phosphorylation” (Figure 5; Figure S7A), GO_CC terms “extracellular region,” “plasma membrane,” and “integral component of membrane” (Figure 5; Figure S7B; Data S9 and S10), and GO_MF terms like “kinase activity” and “ATP binding” (Figure 5; Figure S7C; Data S11 and S12) do not align with this pattern. The effects of treatment with PSY were more similar to that of flg22 and WCS417 treatments in terms of upregulating associated genes linked to these functional terms, whereas PSK downregulated them. This indicates the existence of mechanistic differences among the response pathways to these four stimuli.

### Several basic region/leucine zipper motif (bZIP) TFs are also shared by PSK, PSY, flg22, and WCS417 but display different regulatory patterns from WRKY TFs

Besides WRKY TFs as the most commonly shared TFs that were enriched from DEGs of PSK, PSY, flg22, and WCS417 treatments, several bZIP TFs were also common TFs shared by these treatments (Figure S10). However, unlike the regulatory effects of WRKY TFs, these shared bZIP TFs were primarily enriched from PSK‐upregulated DEGs and PSY‐, flg22‐, and WCS417‐downregulated DEGs (Figure S11). The bZIP TFs are functionally related to plant biotic and abiotic stresses (Dröge‐Laser et al., 2018), suggesting PSK signaling is also involved in these plant responses via bZIP TFs, but exhibiting a different regulatory pattern from WRKY TFs.

## DISCUSSION

In this study, we used a common approach for studying signaling: treatment of whole tissues or plants with bioactive peptide ligands. Furthermore, we used a genotype that lacked endogenous PSK. Although the approach might have resulted in responses from more cells than would naturally occur under physiological conditions, it had the advantage of being able to eliminate interference from endogenous PSK as may happen if WT plants were used. Previously, it was established that PSK generally promotes plant growth while attenuating plant defense mechanisms in some cases, and PSKR1 plays an important role in mediating the trade‐off between plant growth and defense in the rhizosphere microbiome (Matsubayashi et al., 2006; Mosher et al., 2013; Song et al., 2023; Yu et al., 2016). Our transcriptomic analysis offers novel insights into PSK's influence on tipping the balance of plant growth–defense trade‐off. The suppression effect of PSK on defense‐related genes is more predominant in transcriptomics than its activation effect on growth‐related genes. Considering that significantly fewer genes and functional terms are linked with growth promotion compared to defense suppression, it is plausible that some of PSK's growth‐promoting effects may stem from the downregulation of plant defense responses. The defense suppression effect may conserve energy and diminish non‐essential defense activities to prioritize plant growth, especially during the early development stage of Arabidopsis. It is also possible that growth terms are less well defined than defense terms, so the conclusions made here may change as more is known about genes contributing to growth control.

PSK antagonizes the effects of flg22 at multiple different levels. However, PSK treatment does not reduce FLS2 protein levels. Similarly, an early response to flg22, the ROS burst mediated by plasma membrane RBOHD (Kadota et al., 2015), is mostly unaffected by PSK treatment. In contrast, other branches of the flg22 immune response, MAP kinase phosphorylation (which indicates MAP kinase activation) and callose deposition, are reduced by PSK treatment. Differential effects on immune response branches are not uncommon and may reflect different subcellular locations of FLS2 signaling pools (Mbengue et al., 2016; Smith et al., 2014).

The MAP kinase cascade directly phosphorylates WRKY TFs to activate them during plant immune signaling in response to PAMPs (Javed & Gao, 2023; Lassowskat et al., 2014). Interestingly, 48 WRKY TFs are substrates of MPK phosphorylation *in vitro* (Sheikh et al., 2016). Reduced phosphorylation of MPK3 and MPK6 supports our transcriptomics analysis, which shows opposite regulation of WRKY family TFs and their targets by PSK and flg22. Despite different effects on distinct branches of immune signaling, PSK is predicted to interfere with transcriptional reprogramming in response to flg22, since MPK phosphorylation and callose deposition are attenuated in response to flg22. This in turn may permit the greater allocation of resources to growth versus defense.

How might PSK diminish flg22‐induced callose deposition? PSK does not significantly reduce the expression levels of *PMR4*, the pathogen‐induced specific callose synthase (Figure S3). This is consistent with the previous finding that the gene expression levels of callose synthase family genes are only moderately changed in response to various stresses (Ellinger & Voigt, 2014). Instead, PSK downregulates the expression of *ATL31*, a membrane‐associated ubiquitin ligase that is involved in callose deposition during defense responses (Sanmartín et al., 2020). *ATL31* is upregulated in response to flg22 (Figure S3), and its overexpression enhances callose deposition (Maekawa et al., 2012). PSK may counteract flg22's effect on *ATL31* which promotes callose deposition. The expression of *ATL31*'s upstream regulators *WRKY18*, *40*, and *33* are also downregulated by PSK, possibly as a result of reduced phosphorylation level of MPK3/6 mediated by PSK, since MPK3/6 phosphorylates WRKY18 and 33, and WRKY18 and 40 interact with each other (Wang et al., 2018, 2023; Xu et al., 2006). This is further supported by the observation that plants with reduced MPK3 phosphorylation activity have a reduced level of callose deposition (Frei Dit Frey et al., 2014). PSK also downregulates genes associated with the hormones salicylic acid and abscisic acid (Figure S7A), which positively contribute to callose deposition (Wang et al., 2021). This downregulation may constitute an additional indirect mechanism to reduce callose deposition.

**Figure 8:**
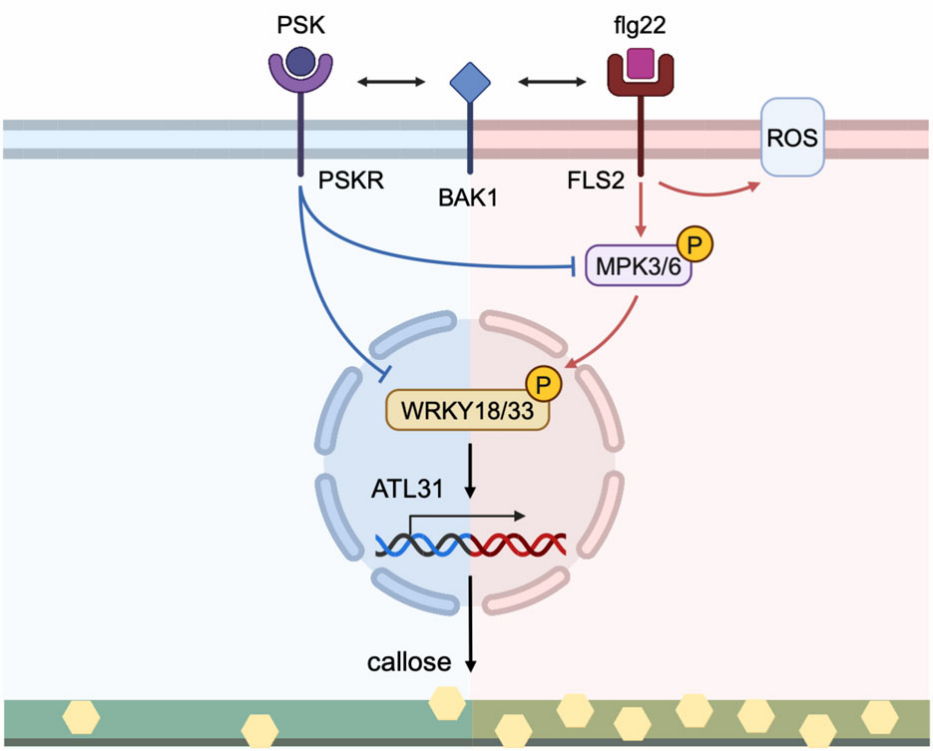
A simplified and speculative model of PSK and flg22 antagonism. PSKR may compete with FLS2 for binding to BAK1. PSK reduces multiple flg22‐induced responses, including the phosphorylation level of MPK3/6, the expression level of *WRKY18/33* and *ATL31*, and the density of callose deposition. However, PSK does not affect ROS level. WRKY TFs shown are based on known MPK targets (Wang et al., 2018, 2023) and the potential regulator of *ATL31* (Birkenbihl et al., 2017) from current literature, but other WRKY TFs may also be involved. A line with a single arrow (red or black) represents a promoting response, a blue line with a perpendicular bar indicates an attenuating effect, and a line with double arrows denotes a co‐receptor relationship. Created in BioRender. Liu, D. (2024) BioRender.com/z39j606.

A simplified and speculative model based on our results and the current literature for antagonism between PSK and flg22 responses is shown in Figure 8. In the model, PSK attenuates various flg22‐induced effects, including MPK phosphorylation, the expression of *WRKY* TF genes and defense genes, and callose deposition. These effects are observable within a short span of 7 min to a longer duration of 48 h. An aspect we did not explore is possible competition between the PSK receptor PSKR1 and the flg22 receptor FLS2 for binding to their common co‐receptor kinase BAK1 (Ladwig et al., 2015; Sun et al., 2013). Although it is possible such competition exists, the very early ROS response, for which BAK1 is required (Chinchilla et al., 2007; Heese et al., 2007), is unaffected. We focused on WRKY18 and WRKY33 due to their extensive documentation and substantial experimental data supporting their roles. The *ATL31* gene serves as a testable driver of phenotypic outcome to illustrate one potential mechanism by which PSK affects callose deposition. This simplified model aims to provide insight into how PSK influences plant defense through WRKY TFs and downstream genes. However, this focus does not diminish the potential significance or roles of other WRKY TFs in Arabidopsis, which may also be important but are not yet as well studied, nor does it exclude other crucial genes affecting callose deposition.

The *tpst* mutant used for transcriptional profiling lacks PSK as well as other sulfated peptides that may interact in some unknown way with the PSK pathway. We accepted this compromise because it was the only way currently available to control PSK application to initiate signaling without the possible obscuring effects of background PSK signaling. The major new impact we detected of the loss of one or more other sulfated peptides is the tuning of the amplitude of flg22‐induced defenses without changing the suppressive effect of PSK on defense readouts measured in both WT and *tpst*. We did not observe higher basal levels of callose or globally higher defense‐related gene expression in *tpst* plants, thus the plants were not constitutively autoimmune. However, FLS2 protein levels are higher in the *tpst* plants than in WT (Figure S8D). This may explain why ROS, MPK phosphorylation, and callose levels in response to flg22 in *tpst* plants are higher than in other genotypes tested (Figures 6 and 7; Figure S8A,B). However, FLS2 protein levels and flg22‐induced ROS are similar in *pskr1 pskr2* and WT plants (Figure S8A,D). Thus, it seems unlikely that loss of PSK signaling can explain FLS2 protein levels in *tpst* plants. Rather, a different TPST substrate peptide such as PSY or another sulfated peptide(s) may repress basal FLS2 levels.

Notably, in response to flg22, *pskr1 pskr2* hydroponic seedlings show lower MPK3/6 phosphorylation and callose deposition levels compared to WT plants. Reduced callose is similarly seen in plants lacking a receptor (RGFR1) for the sulfated peptide root growth factor and other RLKs that are associated with the immune regulator ACD6 (Zhang et al., 2017). We speculate that multiple RLKs (including the PSK receptors) contribute to membrane signaling complexes needed for one or more responses to flg22. In contrast, adult soil‐grown *pskr1 pskr2 psy1* plants were reported to display increased callose with flg22 relative to that seen in wild type (Mosher et al., 2013). Different signaling outcomes may be due to the loss of multiple receptor types at one time and/or the use of different growth conditions.

In terms of their regulatory effects on WRKY TFs and plant defense‐related genes, the TPST‐sulfated PSK and PSY peptides induce changes that are more similar when compared with the effects of flg22 and WCS417 bacterium. This alignment is consistent with the number of DEGs in opposite directions (Figure S6). However, PSK diverges from PSY in its effects on genes associated with protein phosphorylation and kinase activity (Figure 5; Figure S7). PSY's effects are more similar to those of flg22 and WCS417 than to PSK in these functional terms. This suggests that unknown mechanisms may underlie the signaling perception and activation processes of PSK and PSY, and PSY might have different effects on MPK phosphorylation. One example is the pattern‐triggered immunity marker gene *FRK1*. PSK downregulates *FRK1* expression, while PSY, flg22, and WCS417 all upregulate its expression (Figure S5B). Moreover, concerning genes associated with GO_BP term “plant epidermis development,” PSK, PSY, and WCS417 all upregulate these genes, whereas flg22 downregulates them. This pattern aligns with the positive effects of PSK, PSY, and WCS417 on plant growth and the highly inhibitory effects of flg22 (Hu et al., 2018; Pieterse et al., 2021; Wu et al., 2014).

Our bioinformatics analysis provides valuable insights into the effects of PSK and connections with other treatments, especially when combined with our physiological measurements that support antagonism between flg22 and PSK treatments using multiple genotypes. However, it is important to acknowledge the inherent limitations of using informatic methods. Databases like gene ontology (GO) infer functional associations primarily based on associative data, which may not always reflect direct causal relationships. Many gene annotations, including those related to pathogen resistance, are often derived from correlations or computational predictions rather than direct experimental evidence. Additionally, these databases can be biased toward well‐studied genes and may not fully capture the complexity of gene functions across different experimental conditions. Similarly, TF enrichment analysis relies on known binding sites and gene networks, which may be incomplete or context dependent. As a result, while these analyses offer a broad overview of potential gene functions and regulatory networks, their findings should be further tested through direct experimental approaches to assess their biological relevance.

## METHODS

### Plant materials and growth conditions

In all experiments, *Arabidopsis thaliana* Columbia‐0 (Col‐0) ecotype and mutants in the Col‐0 background were used. T‐DNA insertion mutants *tpst‐1* (SALK_009847) and double‐mutant *pskr1‐3 pskr2‐1* (cross of SALK_008585 and SALK_024464) lines were obtained from Dr. Margret Sauter's group (Hartmann et al., 2014; Kutschmar et al., 2009; Stührwohldt et al., 2011).

Seeds were sterilized with 75% ethanol for 1 min and 5% sodium hypochlorite with 0.05% Tween‐20 for 5 min. After sterilization, the seeds were washed with distilled water five times before being placed in six‐well cell culture dishes for growth. The liquid growth medium was prepared as 1/2 Murashige and Skoog (MS) basal medium (Sigma‐Aldrich) with 0.05% MES (Sigma‐Aldrich), 1% sucrose (Fisher Scientific), and 0.1% MS vitamin solution (Sigma‐Aldrich), pH adjusted to 5.7. The dishes were supplemented with 5 mL liquid growth medium per well and were placed horizontally in a growth room with 22°C 16 h light/8 h dark long‐day condition (light intensity at 3000 lux). For ROS assays, plants were grown in soil for 4 weeks at 20°C with 12 h light/12 h dark cycle.

### PSK treatments and RNA extractions

Three independent biological replicates were used in RNA extraction and RNA‐seq. PSK was synthesized by Synbio Technologies and Biomatik, and the lyophilized peptide was dissolved in water and stored at −80°C as 2.3 mM stock solutions. In whole‐seedling profiling, PSK treatment was conducted by adding PSK solution to the growth medium of the experimental *tpst* plants to achieve a final PSK concentration of 100 nM or 1 μM for 5 h. WT and *tpst* control plants were mock treated by adding an equivalent volume of water to the growth medium. In tissue‐specific profiling, PSK treatment was performed by replacing the growth medium of the experimental *tpst* plants with fresh medium containing 10 nM PSK for 30 min, 2 h, and 5 h, or germinating experimental *tpst* plants with medium containing 10 nM PSK for 11 days. WT and *tpst* control plants remained untreated, with no medium replacement. Total RNA was extracted from 30 seedlings per replicate at day 7 in whole‐seedling profiling, or from 18 seedlings with shoot and root separated at day 11 in tissue‐specific profiling, using RNeasy Plant Mini Kit (Qiagen) and the RNase‐Free DNase Set for on‐column DNase digestion (Qiagen).

### RNA‐seq experiments and data analysis

Purified RNA samples were sent to Novogene for poly(A) enrichment mRNA library preparation and sequencing. Twenty million 150‐bp paired‐end reads per sample were generated with the Illumina NoveSeq 6000 System. The obtained reads were mapped to *A. thaliana* TAIR10 genome release (ensemblplants_arabidopsis_thaliana_tair10_gca_000001735_1) (Lamesch et al., 2012) using HISAT2 (v2.0.5) (Kim et al., 2019). FeatureCounts (v1.5.0‐p3) was used to count the read numbers mapped of each gene and FPKM (the expected number of fragments per kilobase of transcript sequence per millions base pairs sequenced) was used to quantify and estimate gene expression (Mortazavi et al., 2008).

The differential expression analysis was performed with the R package DESeq2 (v1.20.0) (Love et al., 2014). The expression levels of each sample were compared to those of the untreated or mock‐treated *tpst* control. The functional enrichment analysis was conducted at the DAVID Knowledgebase (https://david.ncifcrf.gov/) that integrates GO_BP, gene ontology cellular component (GO_CC), and gene ontology molecular function (GO_MF) databases (Huang et al., 2009). The TF enrichment analysis was carried out at PlantRegMap (http://plantregmap.gao‐lab.org/) using the FunTFBS method (Tian et al., 2020). The heatmaps were generated using the R package ComplexHeatmap (v2.13.1) with rows split by K‐means clustering and distance measured by predefined Pearson method (Gu et al., 2016). The Venn diagrams were created with Venny 2.1.0 (Oliveros, 2007).

### ROS assay

ROS induction by flg22 was assayed and quantified as described (Tateda et al., 2014). flg22 was synthesized by Biomatik, and the lyophilized peptide was dissolved in water and stored at −80°C as 100 μM or 1 mM stock solutions. Leaf discs (12 discs per genotype/treatment in each experiment) were prefloated on water or 1 μM PSK overnight, and total ROS accumulation was measured by chemiluminescence after adding 1 μM flg22. Alternatively, leaf discs were treated with PSK for shorter time (15 min to 5 h). To compare different experiments, ROS in WT + flg22 was set as 1. Relative ROS accumulation was quantified from at least four experiments for each PSK treatment.

### Protein isolation and immunoblot analysis

11‐day‐old seedlings grown in six‐well cell culture dishes (3–5 seedlings per sample) or leaf discs (5–8 discs per sample) from soil‐grown plants were treated with 1 μM or 200 nM PSK overnight and MAP kinases were activated by adding 1 μM or 200 nM flg22, respectively, for 7–15 min. Alternatively, plant samples were treated with PSK for shorter time (10–40 min). Total proteins were isolated by grinding tissue in cold isolation buffer (Tris HCl 150 mM pH 7.5, NaCl 150 mM, EDTA 5 mM, DTT 1 mM, protease inhibitor for plants [Sigma], phosphatase inhibitor [Pierce/Thermo Fisher], and Triton X‐100 1%). Proteins were resolved by SDS‐Polyacrylamide Gel Electrophoresis (SDS‐PAGE), transferred to a Polyvinylidene Fluoride (PVDF) membrane, and probed with following rabbit antibodies: phosphorylated MPK antibody phospho‐p44/42 MAPK (Erk1/2) (Thr202/Tyr204) [Cell Signaling Technology, 1:1000–1:2000]; MPK3 antibody [Sigma, 1:2000]; FLS2 antibody [1:500, (Chinchilla et al., 2007)]; and anti‐rabbit‐HRP secondary antibodies (Thermo Fisher). Proteins were detected by chemiluminescence using Bio‐Rad ChemiDoc XRS+ imager. Relative band intensities were quantified from at least four experiments using Image Lab software (Bio‐Rad) and normalized relative to total protein from Coomassie blue‐stained membranes, similar to a previously described method (Tateda et al., 2014). Different treatment durations and peptide concentrations gave similar results, and the experiments were analyzed together by setting immunoblot signal in WT without PSK to 1.

### Callose deposition

Plants grown in six‐well cell culture dishes (five seedlings per well) were treated with water or PSK solutions at day 7 for 24 h, and then treated with water or flg22 solutions at day 8 for 24 h. The treatments were conducted by adding PSK solution and flg22 solutions to the liquid 1/2 MS medium to achieve a final concentration of 100 nM and 1 μM, respectively. The plants were fixed and stained with aniline blue as described (Mason et al., 2020). Callose deposits were quantified with Fiji using images taken with a Leica DMR fluorescence microscope (10× objective). At least 52 cotyledons with high‐quality images from 30 seedlings per genotype and per treatment were used for analysis.

## AUTHOR CONTRIBUTIONS

DL, JJ, JMM, and JTG designed the experiments. DL performed the experiments, conducted bioinformatic analysis, and analyzed data. JJ performed the MPK phosphorylation and FLS2 quantification experiments and analyzed ROS data. JMM performed the ROS experiment. DL, JJ, and JTG wrote the manuscript.

## Supporting information


**Figure S1.** Synthetic PSK enhances the root growth of hydroponic *tpst* plants.
**Figure S2.** Heatmaps of GO_BP terms enriched from PSK‐induced DEGs.
**Figure S3.** Heatmap of representative differentially expressed genes with PSK, PSY, flg22, and WCS417 treatments.
**Figure S4.** WRKY TFs in ATRM map.
**Figure S5.** Heatmaps of gene expression in ATRM map.
**Figure S6.** Heatmap of the percentage of DEGs regulated in the opposite direction by PSK, PSY, flg22 and WCS417 among all DEGs, using row category as the base.
**Figure S7.** Heatmaps of all Gene Ontology terms.
**Figure S8.** ROS burst, MPK phosphorylation and FLS2 protein levels are higher in *tpst* plants than in WT.
**Figure S9.** PSK effects on flg22‐ induced callose deposition.
**Figure S10.** Heatmap of all enriched TFs from PSK, PSY, flg22, and WCS417 up‐ and downregulated DEGs.
**Figure S11.** Heatmaps of bZIP TFs enrichment and expression among multiple stimuli.

**Table S1.** Information of each plant sample and PSK treatment in RNA‐seq experiments.
**Table S2.** W‐box motifs in the promoter regions of up‐ and downregulated DEGs.
**Table S3.** W‐box motifs presence in the promoter regions of genes involved in PSK signaling.
**Table S4.** Growth conditions and treatment methods for RNA‐seq data used in the comparison of PSK, PSY, flg22 and WCS417 induced DEGs.

**Data S1.** List of DEGs with different PSK treatments.

**Data S2.** List of enriched GO_BP functional terms from upregulated DEGs in each treatment.

**Data S3.** List of enriched GO_BP functional terms from downregulated DEGs in each treatment.

**Data S4.** List of enriched TFs from upregulated DEGs in each treatment.

**Data S5.** List of enriched TFs from downregulated DEGs in each treatment.

**Data S6.** Overlap of DEGs with PSK and flg22 treatments.

**Data S7.** Overlap of enriched WRKY TFs and differentially expressed WRKY TFs in each treatment.

**Data S8.** List of all enriched and differentially expressed WRKY TFs in each treatment.

**Data S9.** List of enriched GO_CC functional terms from upregulated DEGs in each treatment.

**Data S10.** List of enriched GO_CC functional terms from downregulated differentially DEGs in each treatment.

**Data S11.** List of enriched GO_MF functional terms from upregulated DEGs in each treatment.

**Data S12.** List of enriched GO_MF functional terms from downregulated DEGs in each treatment.

## Data Availability

The RNA‐seq data discussed in this study have been deposited in NCBI's Gene Expression Omnibus (GEO) database and are accessible through GEO series accession number GSE254988 (https://www.ncbi.nlm.nih.gov/geo/query/acc.cgi?acc=GSE254988).
