# Supplementary material for: Phytosulfokine downregulates defense‐related WRKY transcription factors and attenuates pathogen‐associated molecular pattern‐triggered immunity
Source: Plant J. 2024 Dec 11;120(6):2367–84. doi: 10.1111/tpj.17115 (PMC11658183; doi:10.1111/tpj.17115)
Supplement: Supplementary file 1 — Figure S1. Synthetic PSK enhances the root growth of hydroponic tpst plants. Figure S2. Heatmaps of GO_BP terms enriched from PSK‐induced DEGs. Figure S3. Heatmap of representative differentially expressed genes with PSK, PSY, flg22, and WCS417 treatments. Figure S4. WRKY TFs in ATRM map. Figure S5. Heatmaps of gene expression in ATRM map. Figure S6. Heatmap of the percentage of DEGs regulated in the opposite direction by PSK, PSY, flg22 and WCS417 among all DEGs, using row category as the base. Figure S7. Heatmaps of all Gene Ontology terms. Figure S8. ROS burst, MPK phosphorylation and FLS2 protein levels are higher in tpst plants than in WT. Figure S9. PSK effects on flg22‐ induced callose deposition. Figure S10. Heatmap of all enriched TFs from PSK, PSY, flg22, and WCS417 up‐ and downregulated DEGs. Figure S11. Heatmaps of bZIP TFs enrichment and expression among multiple stimuli. [file TPJ-120-2367-s003.zip › 3_sup-Figures.pdf]

Supplementary Figure 1

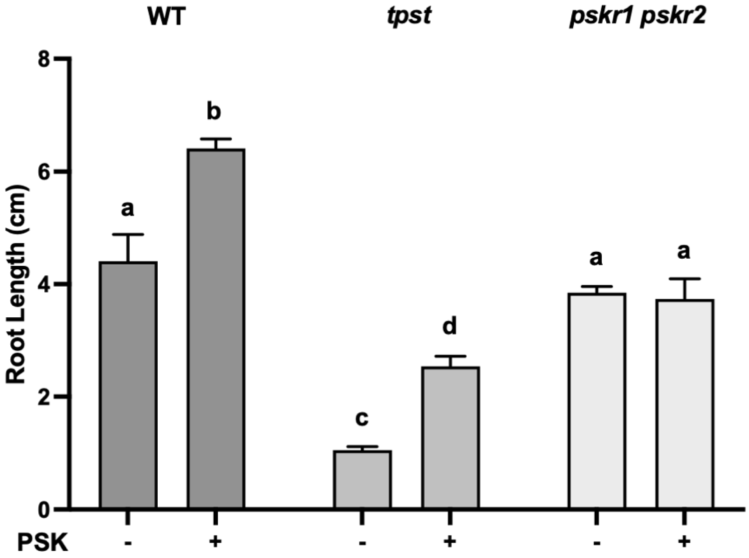

Supplementary Figure 1. Synthetic PSK enhances the root growth of hydroponic *tpst* plants. WT and *tpst* plants were grown in 6-well cell culture dishes with liquid growth medium supplemented with or without 10 nM PSK. The images of plants were taken on the 9th day after vernalization and were subsequently used for root length quantification using Fiji. Different letters indicate significant difference in one-way ANOVA analysis (p-value < 0.05, Fisher's test).

Supplementary Figure 2A

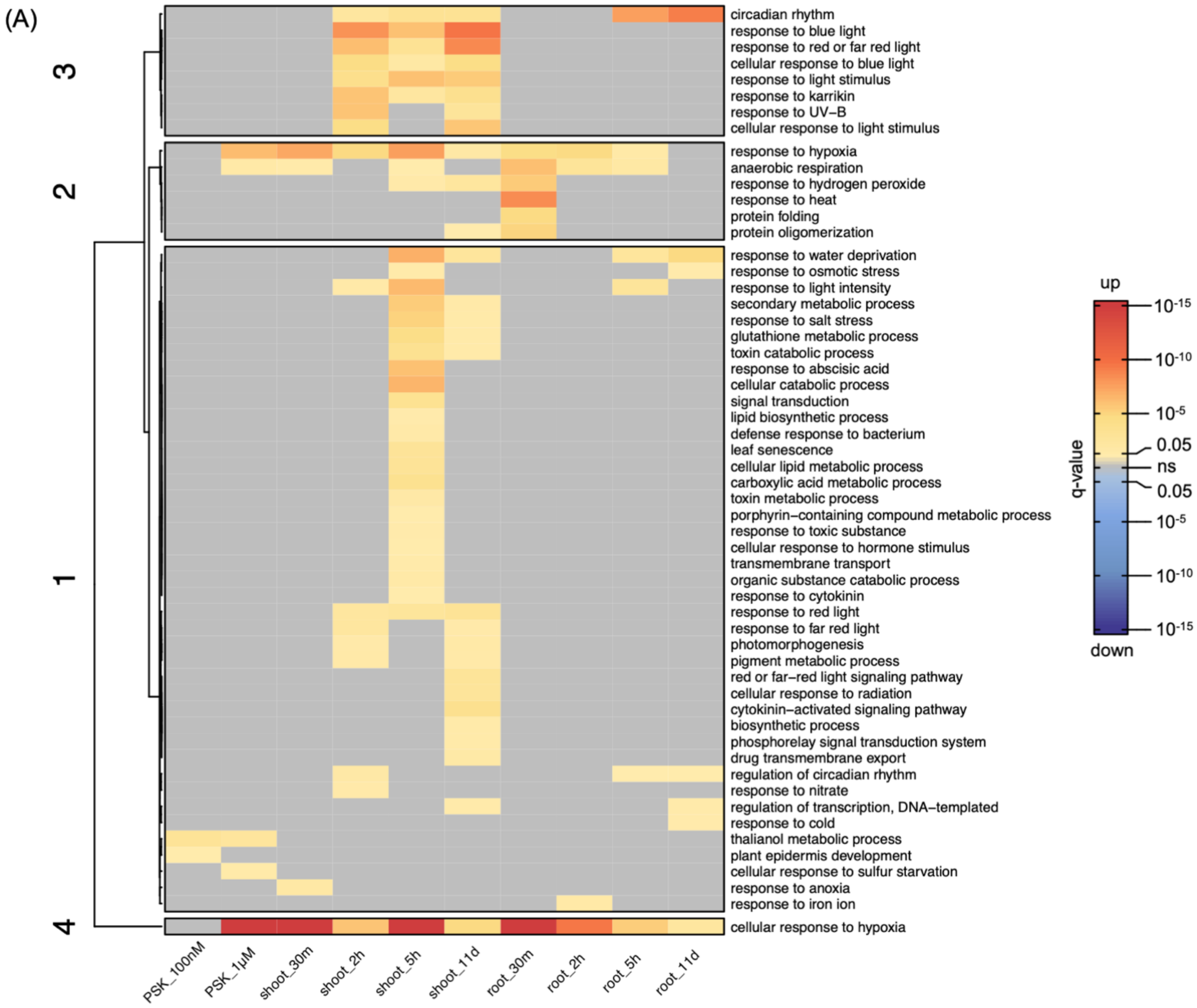

Supplementary Figure 2. Heatmaps of GO_BP terms enriched from PSK-induced DEGs. (A) Heatmap of GO_BP terms enriched from PSK upregulated DEGs. The dendrogram on the side of the heatmap illustrates the hierarchical clustering of rows using k-means clustering with numbers on the branches denoting the distance measures. The intensity of red reflects their significance measured by q-value, utilizing the cut-off of q-value < 0.05. Gray represents data points outside the cut-off criteria.

Supplementary Figure 2B

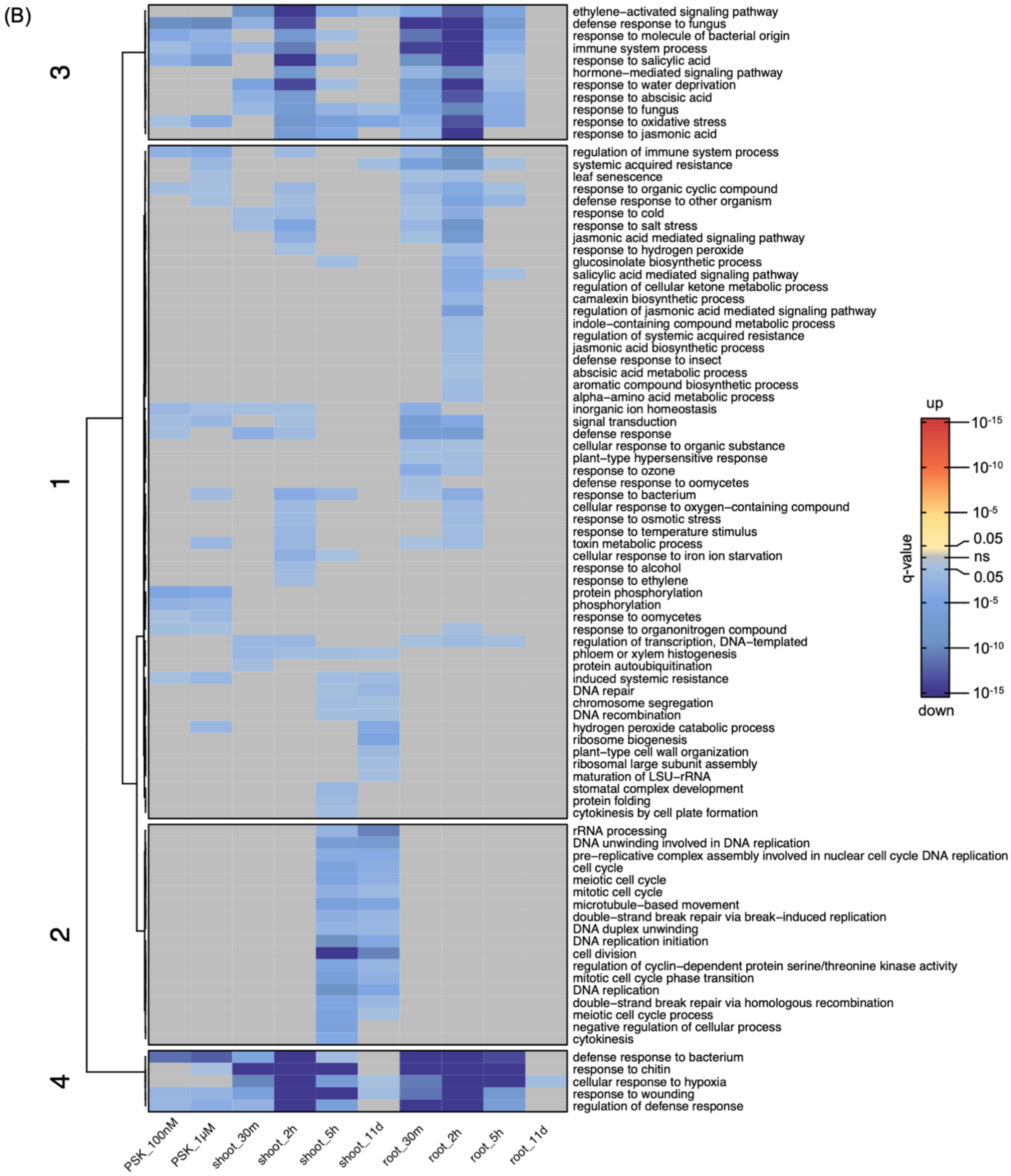

Supplementary Figure 2. (B) Heatmap of GO_BP terms enriched from PSK downregulated DEGs. The dendrogram on the side of the heatmap illustrates the hierarchical clustering of rows using k-means clustering with numbers on the branches denoting the distance measures. The intensity of blue reflects their significance measured by q-value, utilizing the cut-off of q-value < 0.05. Gray represents data points outside the cut-off criteria.

Supplementary Figure 3

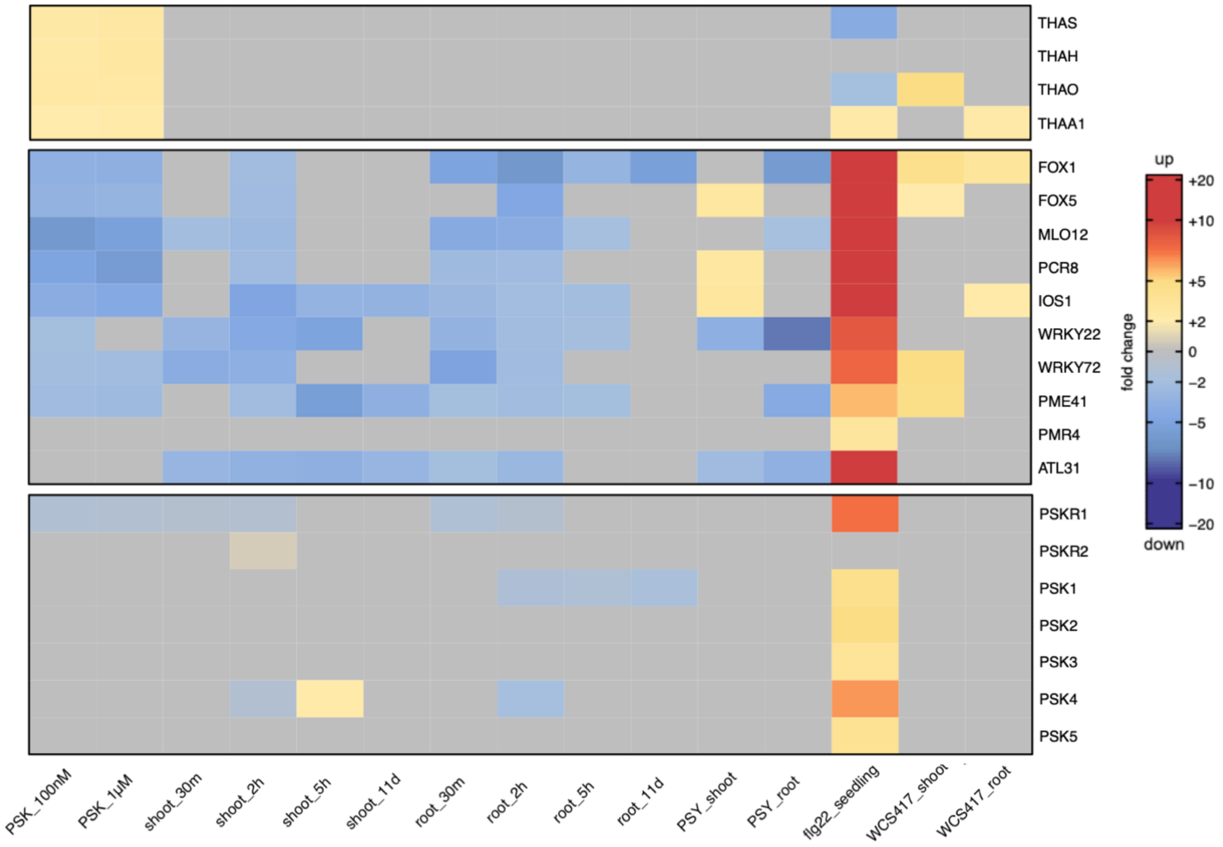

Supplementary Figure 3. Heatmap of representative differentially expressed genes with PSK, PSY, flg22, and WCS417 treatments. Genes in the top section are thalianin pathway-related, genes in the middle section are defense-related, and genes in the bottom section are PSK signaling-related. Red indicates upregulation, while blue indicates downregulation. The intensity of color reflects their fold changes, utilizing the cut-off of ≥ 2-fold change and adjusted p-value < 0.05 for thalianin pathway-related and defense-related genes, and adjusted p-value < 0.05 for PSK signaling-related genes. Gray represents data points outside the cut-off criteria.

Supplementary Figure 4

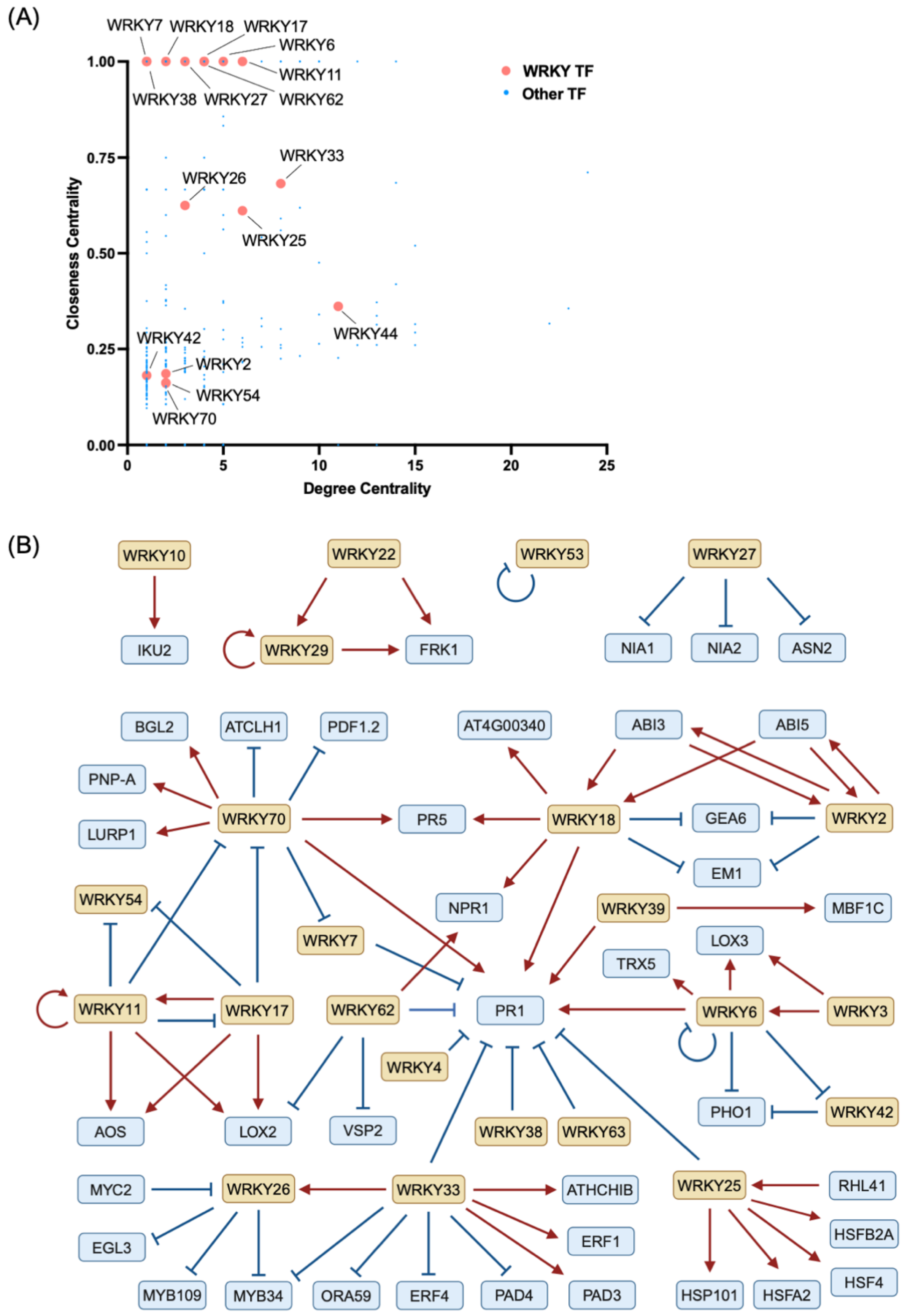

Supplementary Figure 4. WRKY TFs in ATRM map. (A) Degree centrality and closeness centrality of manually curated high-confidence TFs from ATRM. Red dots indicates WRKY TFs, blue dots indicates all other TFs. (B) WRKY regulatory network derived from ATRM. Red arrow indicates activation effect, blue ended-line indicates repression effect. Created in BioRender. Liu, D. (2024) BioRender.com/u13u417.

Supplementary Figure 5

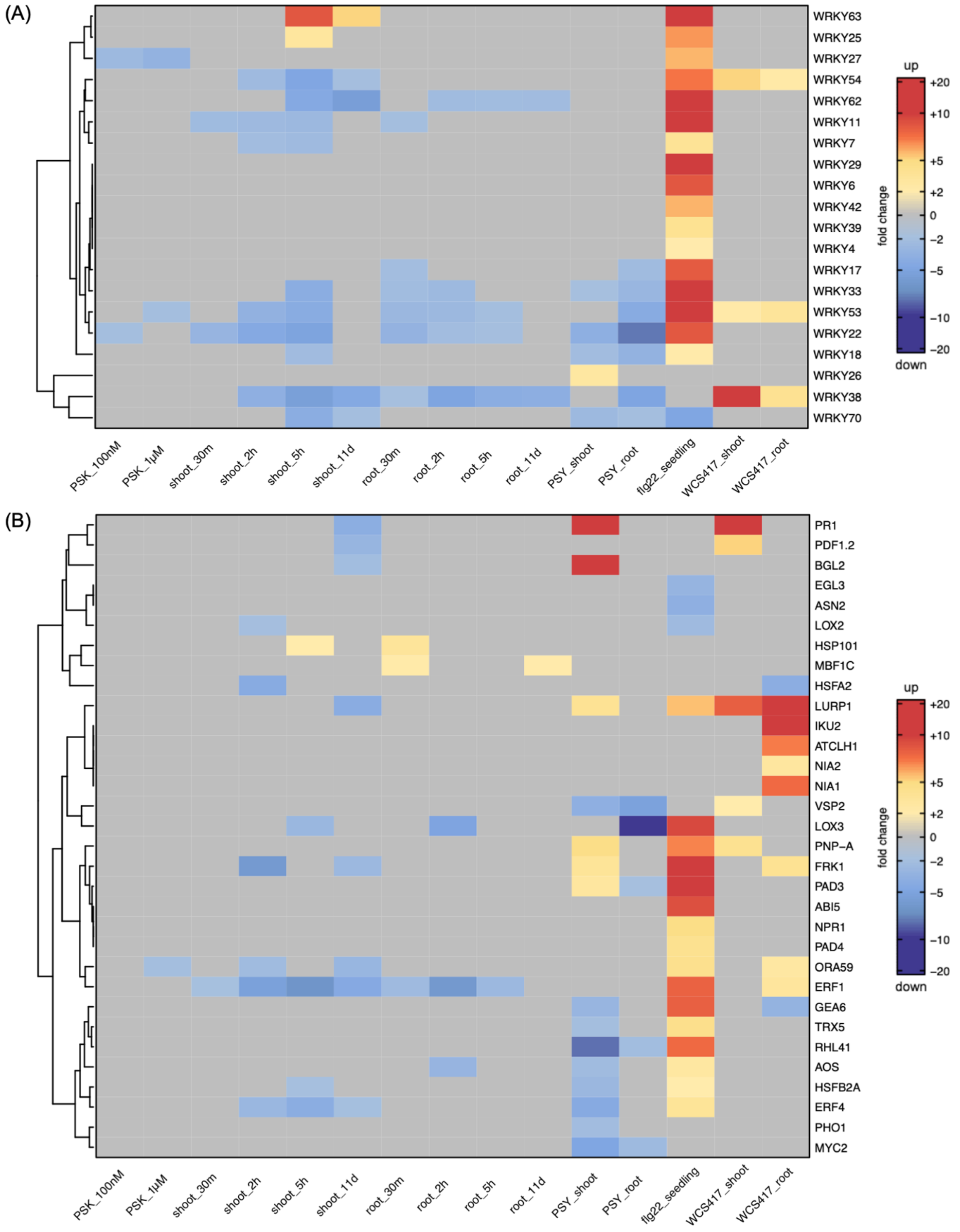

Supplementary Figure 5. Heatmaps of gene expression in ATRM map. (A) Heatmap of representative differentially expressed WRKY TFs in ATRM map with PSK, PSY, flg22, and WCS417 treatments. The dendrogram on the side of the heatmap illustrates the hierarchical clustering of rows using k-means clustering with numbers on the branches denoting the distance measures. Red indicates upregulation, while blue indicates downregulation. The intensity of color reflects their fold changes, utilizing the

cut-off of ≥ 2-fold change and adjusted p-value < 0.05. Gray represents data points outside the cut-off criteria. (B) Heatmap of representative differentially expressed genes downstream of WRKY TFs in ATRM map with PSK, PSY, flg22, and WCS417 treatments. The dendrogram on the side of the heatmap illustrates the hierarchical clustering of rows using k-means clustering with numbers on the branches denoting the distance measures. Red indicates upregulation, while blue indicates downregulation. The intensity of color reflects their fold changes, utilizing the cut-off of ≥ 2-fold change and adjusted p-value < 0.05. Gray represents data points outside the cut-off criteria.

Supplementary Figure 6

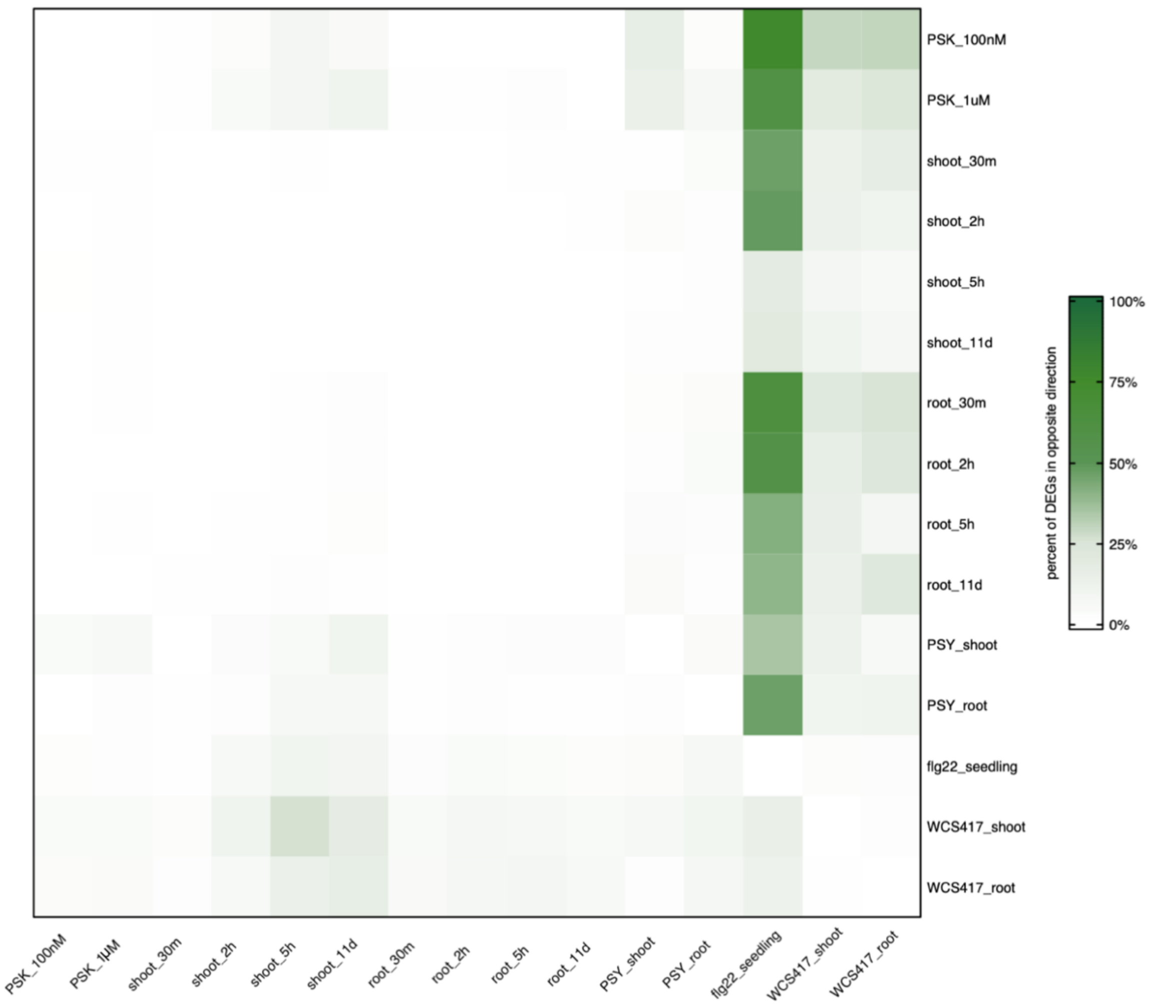

Supplementary Figure 6. Heatmap of the percentage of DEGs regulated in the opposite direction by PSK, PSY, flg22 and WCS417 among all DEGs, using row category as the base. The intensity of green indicates percentage.

Supplementary Figure 7A

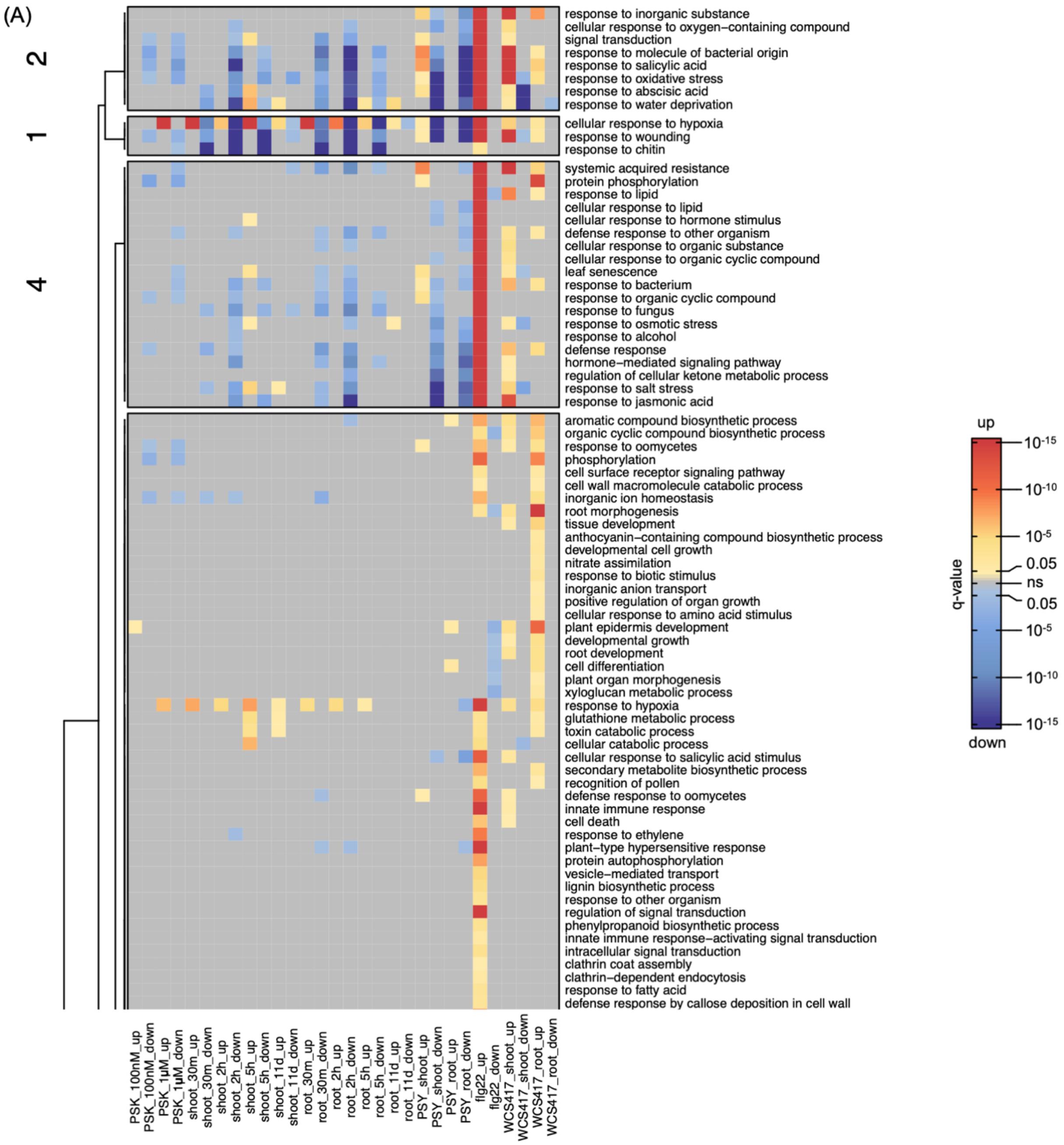

Supplementary Figure 7A, continued

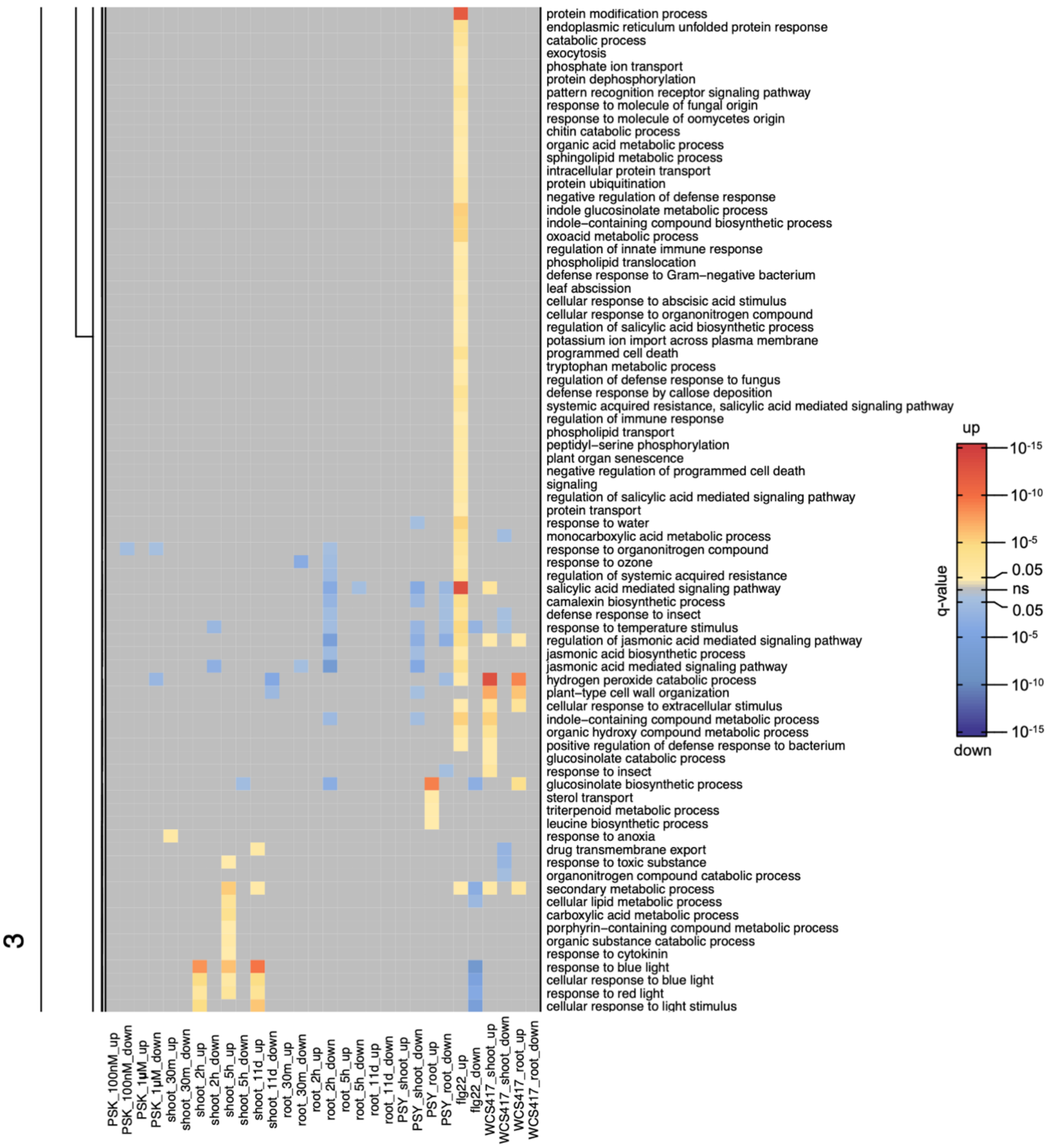

Supplementary Figure 7A, continued

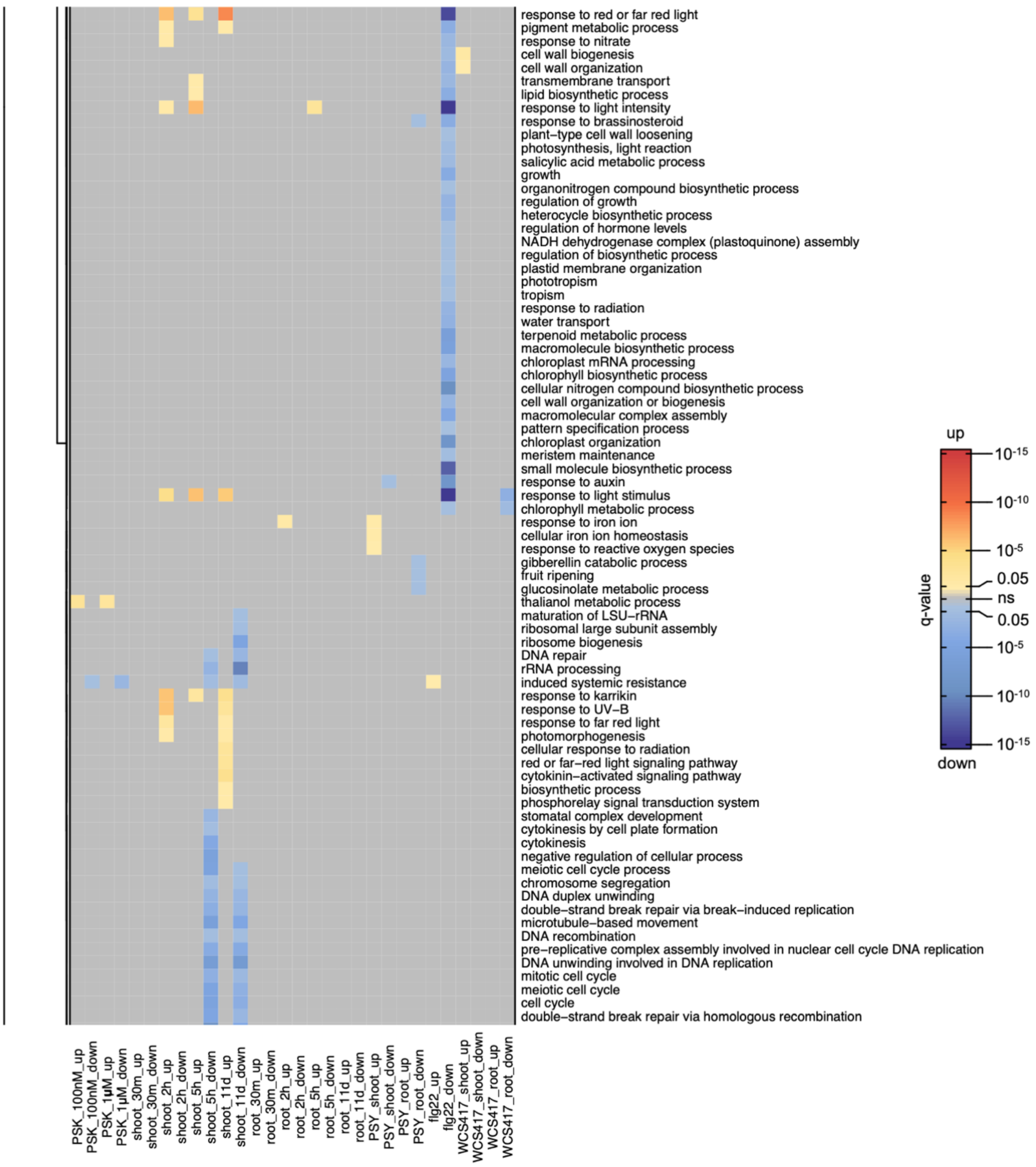

Supplementary Figure 7A, continued

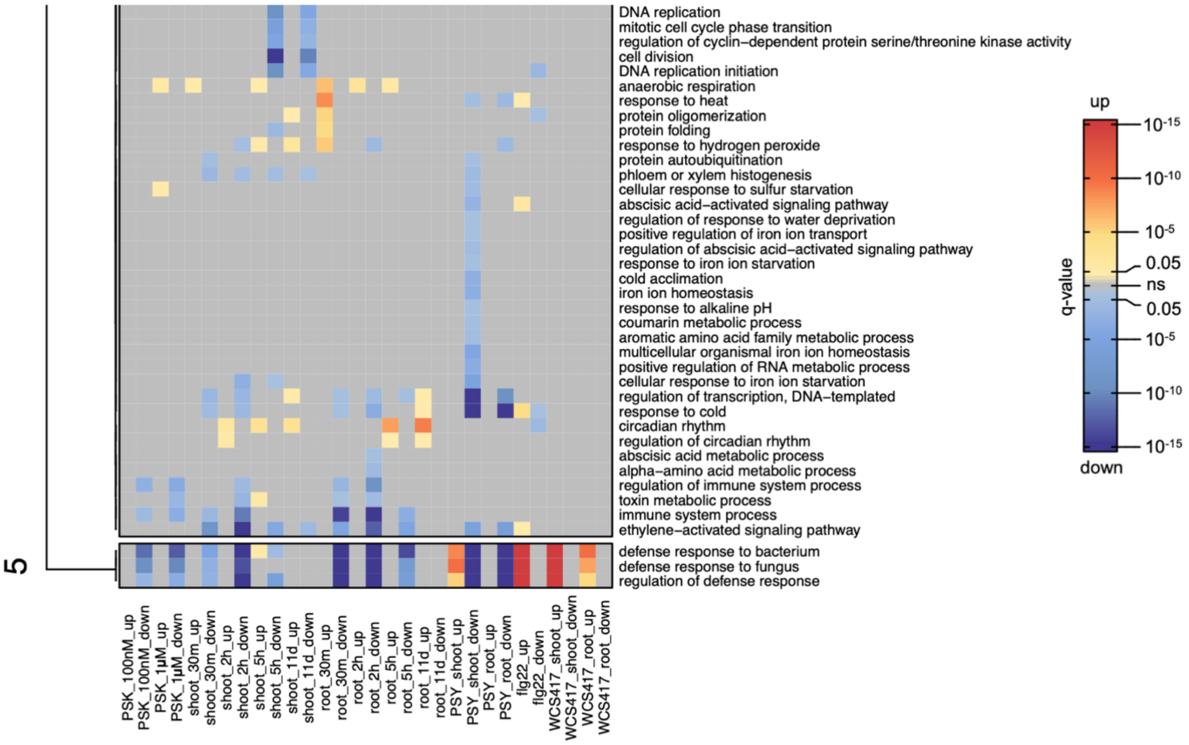

Supplementary Figure 7. Heatmaps of all Gene Ontology terms. (A) Heatmap of all enriched GO_BP terms from PSK, PSY, flg22, and WCS417 treatments. The dendrogram on the side of the heatmap illustrates the hierarchical clustering of rows using k-means clustering with numbers on the branches denoting the distance measures. Red denotes functional terms enriched from upregulated DEGs, while blue denotes functional terms enriched from downregulated DEGs. The intensity of color reflects their significance measured by q-value, utilizing the cut-off of q-value < 0.05. Gray represents data points outside the cut-off criteria.

Supplementary Figure 7B

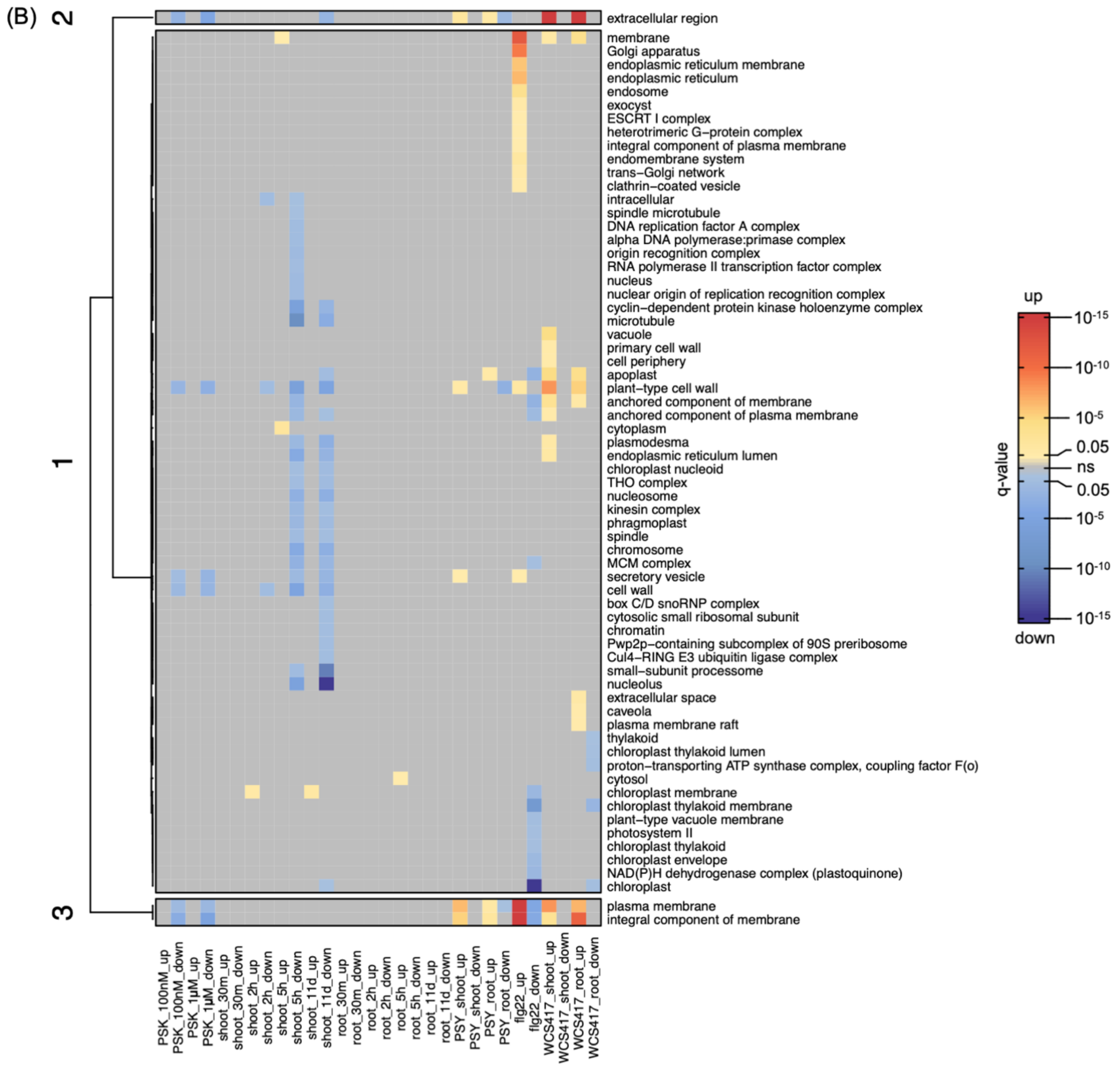

Supplementary Figure 7. (B) Heatmap of all enriched GO_CC terms from PSK, PSY, flg22, and WCS417 treatments. The dendrogram on the side of the heatmap illustrates the hierarchical clustering of rows using k-means clustering with numbers on the branches denoting the distance measures. Red denotes functional terms enriched from upregulated DEGs, while blue denotes functional terms enriched from downregulated DEGs. The intensity of color reflects their significance measured by q-value, utilizing the cut-off of q-value < 0.05. Gray represents data points outside the cut-off criteria.

Supplementary Figure 7C

(C)

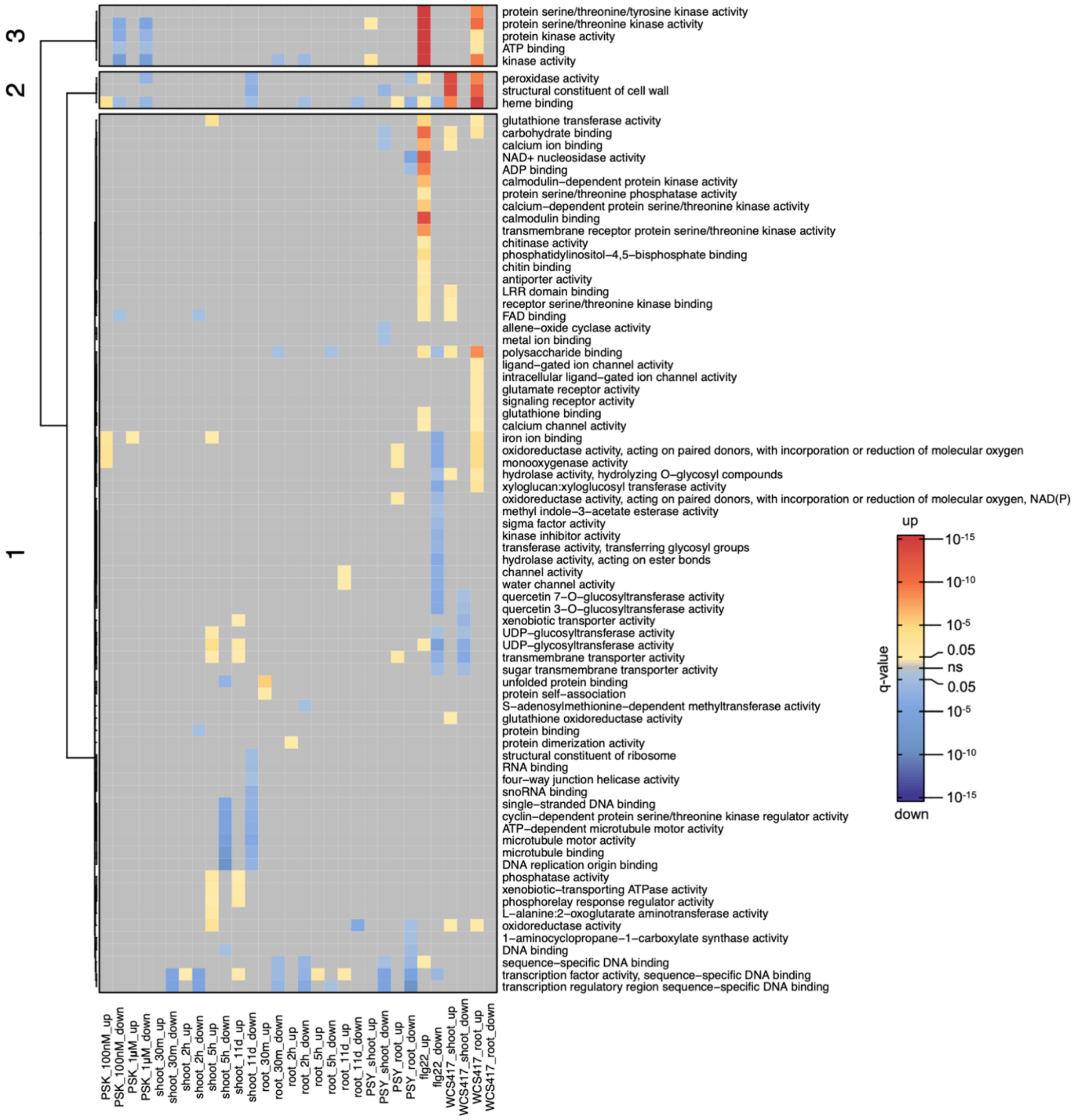

Supplementary Figure 7. (C) Heatmap of all enriched GO_MF terms from PSK, PSY, flg22, and WCS417 treatments. The dendrogram on the side of the heatmap illustrates the hierarchical clustering of rows using k-means clustering with numbers on the branches denoting the distance measures. Red denotes functional terms enriched from upregulated DEGs, while blue denotes functional terms enriched from downregulated DEGs. The intensity of color reflects their significance measured by q-value, utilizing the cut-off of q-value < 0.05. Gray represents data points outside the cut-off criteria.

Supplementary Figure 8

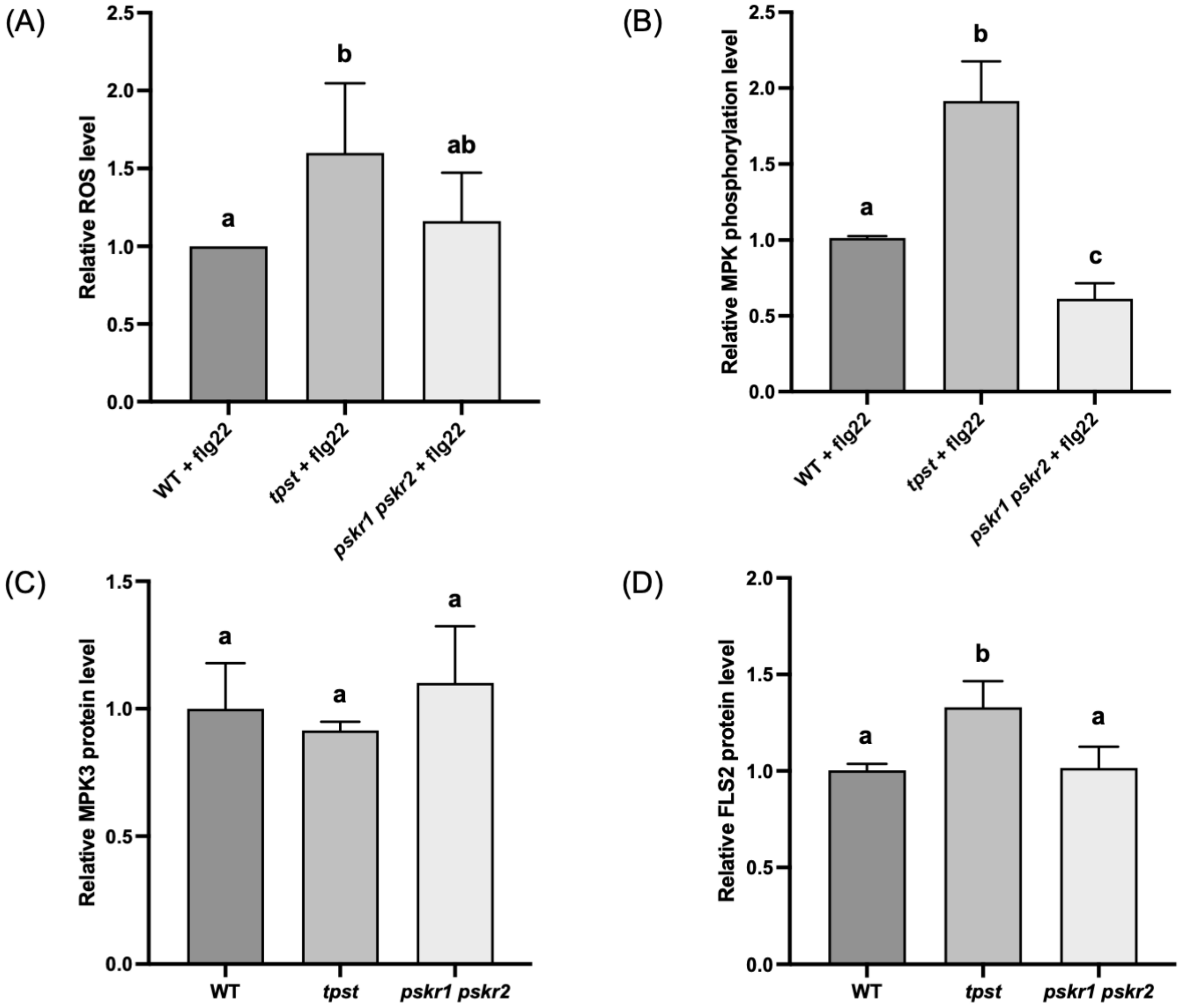

Supplementary Figure 8. ROS burst, MPK phosphorylation and FLS2 protein levels are higher in *tpst* plants than in WT. (A) flg22-induced ROS in different genotypes: *tpst* plants have higher ROS response than WT. (B) flg22-induced MPK3 and MPK6 phosphorylation in different genotypes. The three genotypes shown have different levels of MPK phosphorylation in response to flg22. (C) Relative MPK3 protein level was similar in all genotypes. (D) *tpst* plants have higher level of FLS2 than WT and *pskr1 pskr2*. In all graphs (A-D) different letters indicate significant difference in one-way ANOVA analysis (p-value < 0.05, Fisher's test).

Supplementary Figure 9

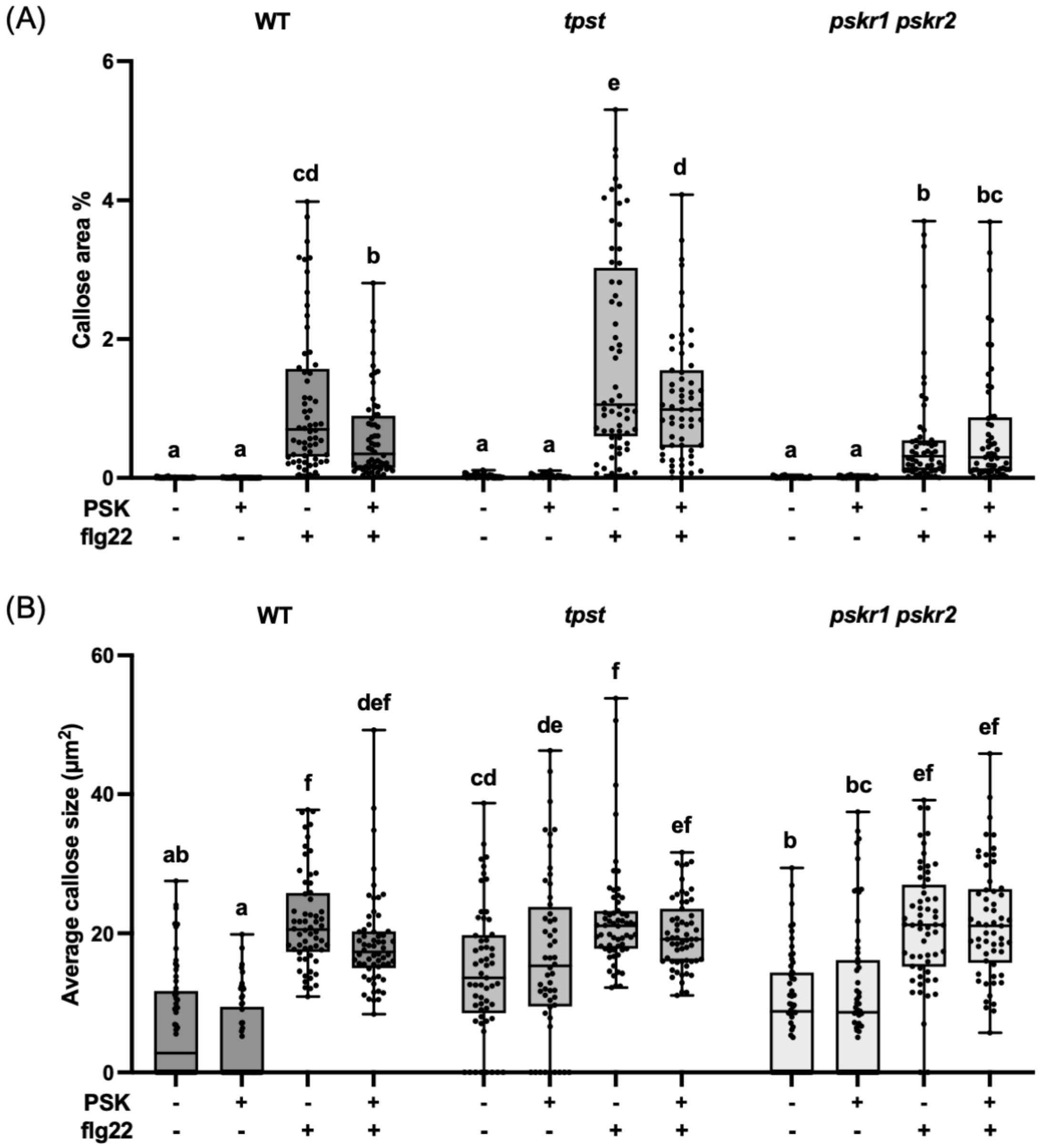

Supplementary Figure 9. PSK effects on flg22-induced callose deposition. (A) Callose area percentage calculated as the area of callose deposits relative to the total plant area in WT, *tpst*, *pskr1 pskr2* plants treated with or without PSK and flg22. Each group contains at least 52 cotyledons with high-quality images from 30 seedlings, which were pooled for analysis. Different letters indicate significant difference in one-way ANOVA analysis (p-value < 0.05, Tukey's test). (B) The average size of callose deposits in WT, *tpst*, *pskr1 pskr2* plants treated with or without PSK and flg22. Each group contains at least 52 cotyledons with high-quality images from 30 seedlings, which were pooled for analysis. Different letters indicate significant difference in one-way ANOVA analysis (p-value < 0.05, Tukey's test).

Supplementary Figure 10

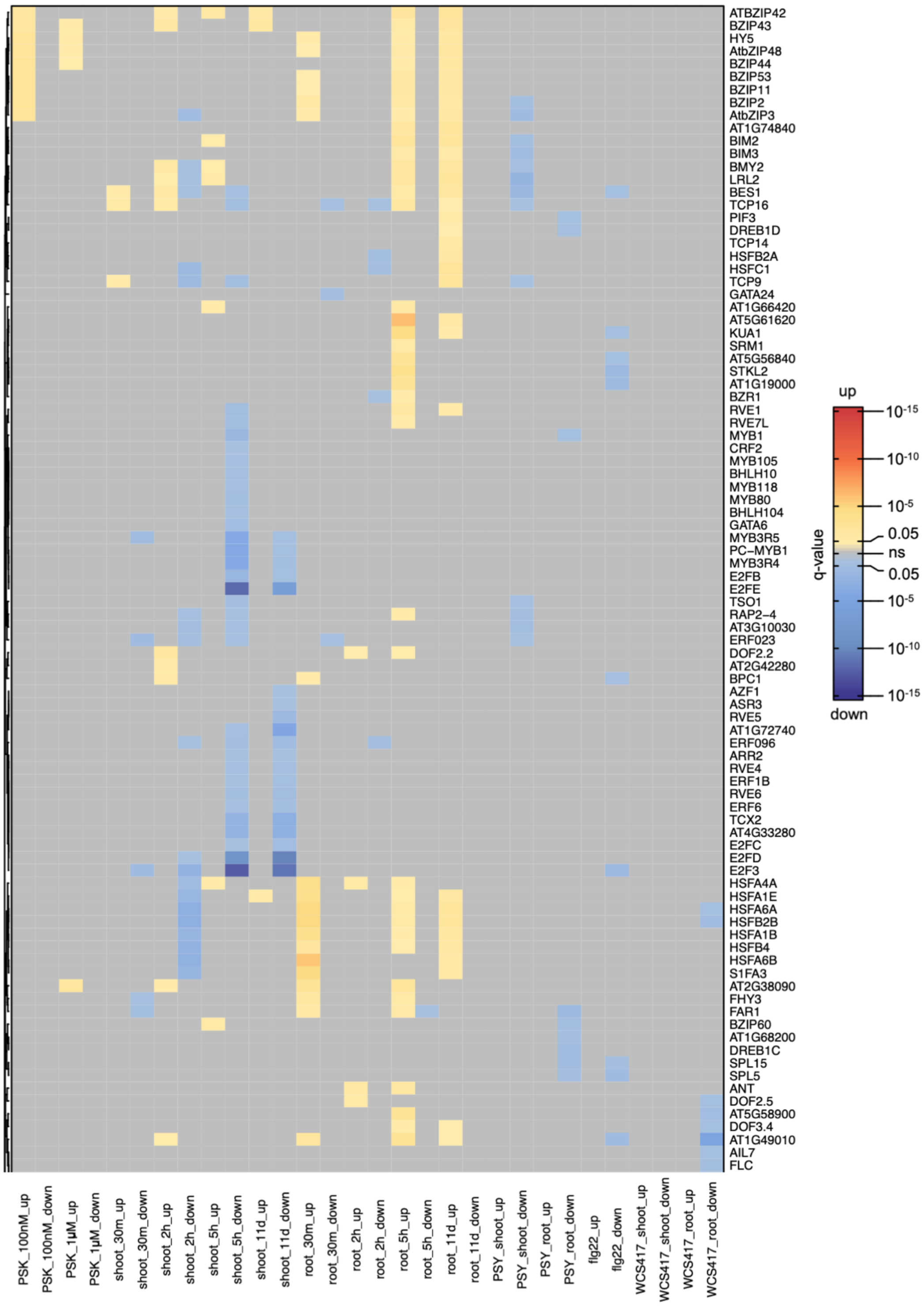

Supplementary Figure 10, continued

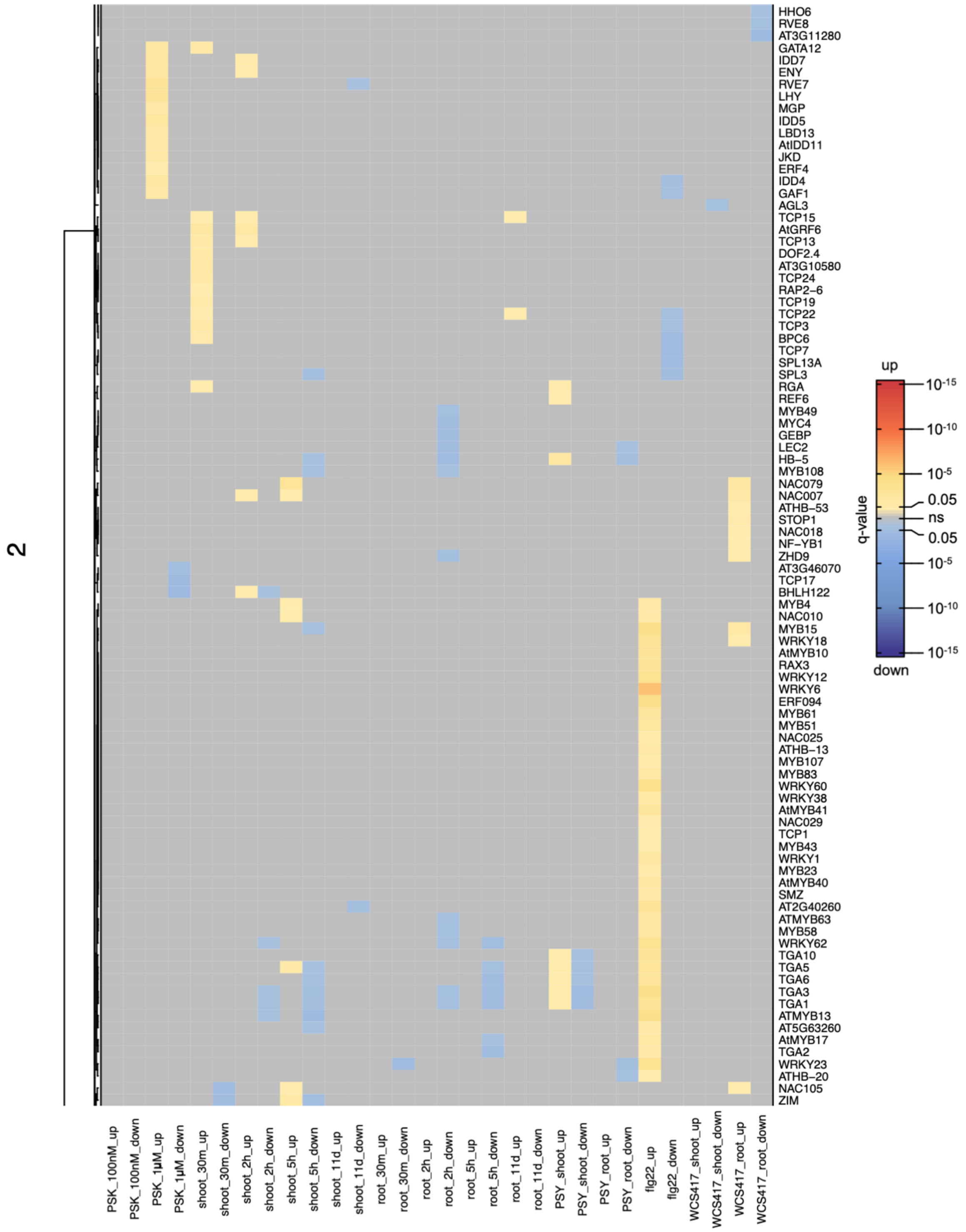

Supplementary Figure 10, continued

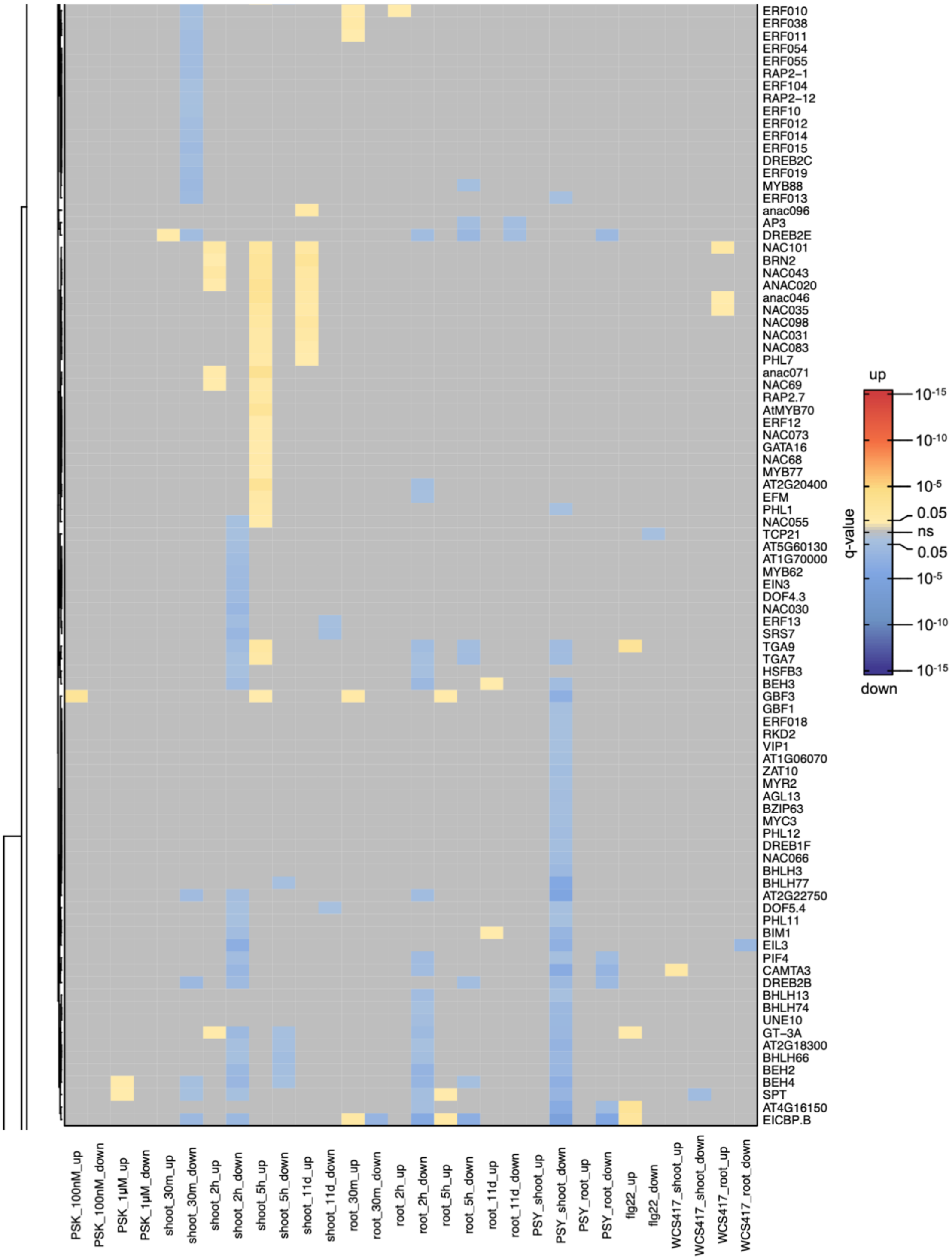

Supplementary Figure 10, continued

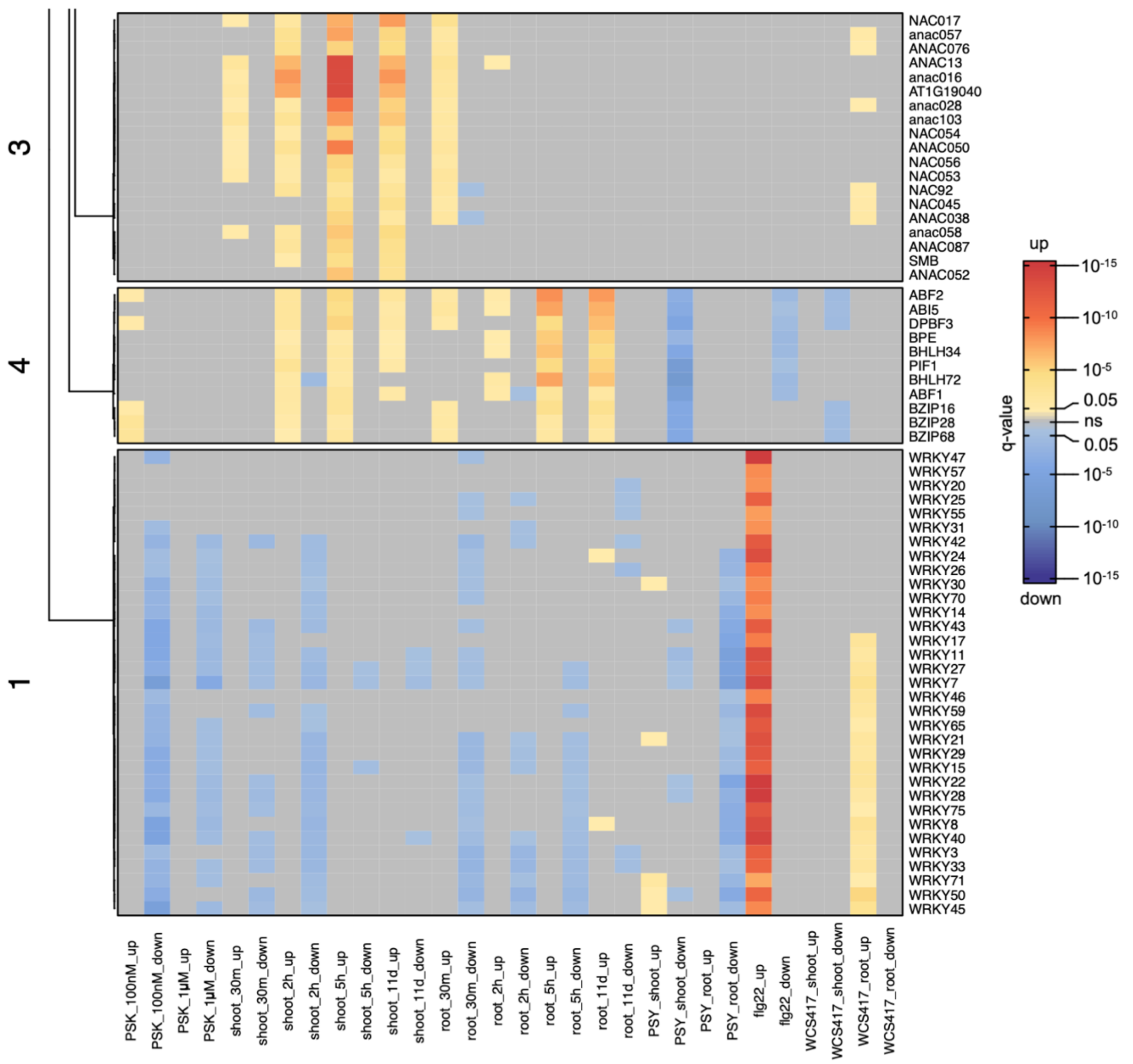

Supplementary Figure 10. Heatmap of all enriched TFs from PSK, PSY, flg22, and WCS417 up- and downregulated DEGs. The dendrogram on the side of the heatmap illustrates the hierarchical clustering of rows using k-means clustering with numbers on the branches denoting the distance measures. Red denotes enrichment from upregulated DEGs, while blue denotes enrichment from downregulated DEGs. The intensity of color reflects their significance measured by q-value, utilizing the cut-off of q-value < 0.05. Gray represents data points outside the cut-off criteria.

Supplementary Figure 11A

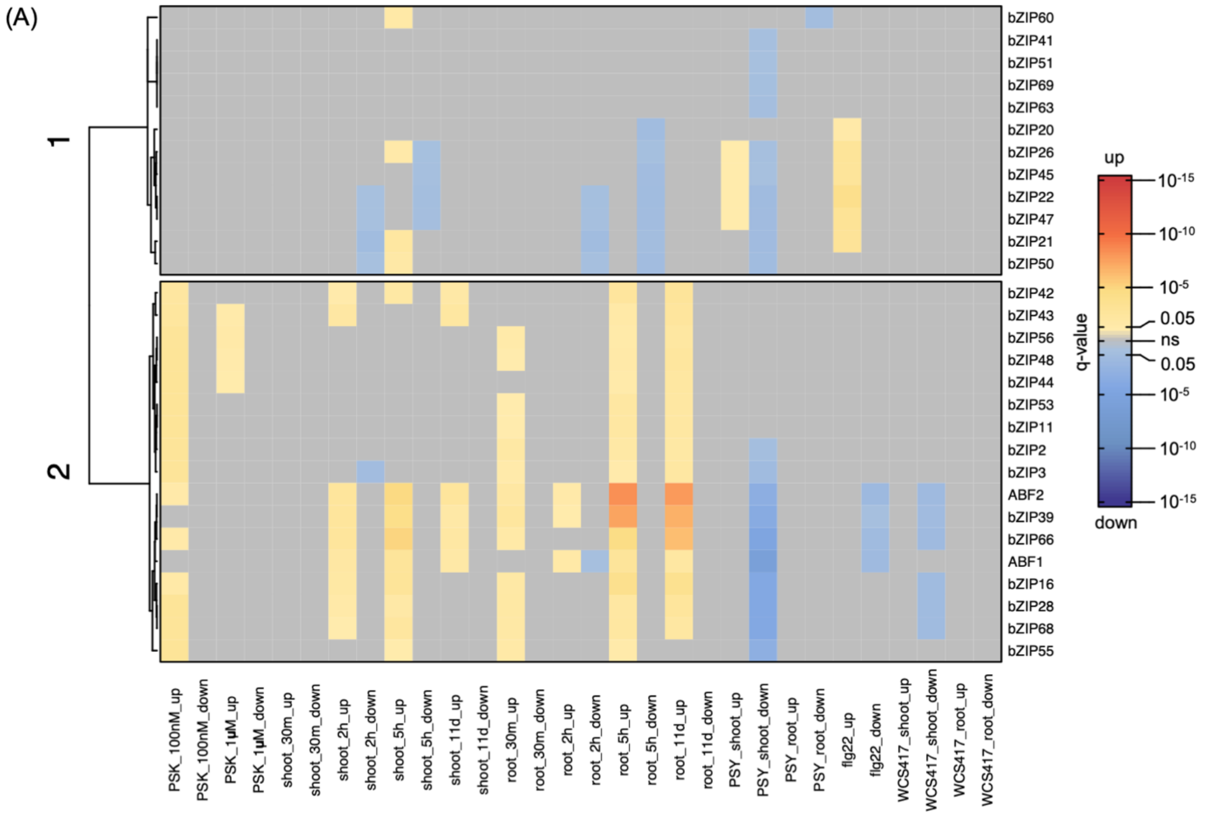

Supplementary Figure 11. Heatmaps of bZIP TFs enrichment and expression among multiple stimuli. (A) Heatmap of enriched bZIP TFs from PSK, PSY, flg22, and WCS417 up- and downregulated DEGs. The dendrogram on the side of the heatmap illustrates the hierarchical clustering of rows using k-means clustering with numbers on the branches denoting the distance measures. Red denotes enrichment from upregulated DEGs, while blue denotes enrichment from downregulated DEGs. The intensity of color reflects their significance measured by q-value, utilizing the cut-off of q-value < 0.05. Gray represents data points outside the cut-off criteria.

Supplementary Figure 11B

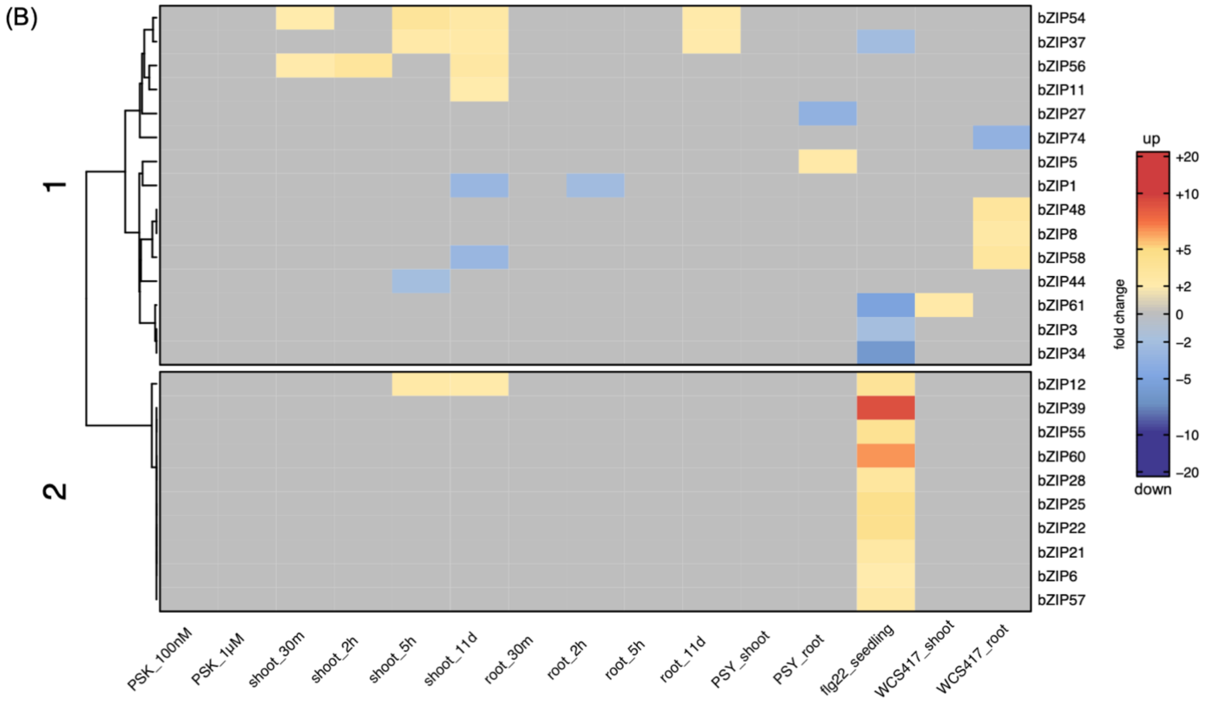

Supplementary Figure 11. (B) Heatmap of differentially expressed bZIP TFs with PSK, PSY, flg22, and WCS417 treatments. The dendrogram on the side of the heatmap illustrates the hierarchical clustering of rows using k-means clustering with numbers on the branches denoting the distance measures. Red indicates upregulation, while blue indicates downregulation. The intensity of color reflects their fold changes, utilizing the cut-off of ≥ 2-fold change and adjusted p-value < 0.05. Gray represents data points outside the cut-off criteria.
