# Supplementary material for: Phytosulfokine downregulates defense‐related WRKY transcription factors and attenuates pathogen‐associated molecular pattern‐triggered immunity
Source: Plant J. 2024 Dec 11;120(6):2367–84. doi: 10.1111/tpj.17115 (PMC11658183; doi:10.1111/tpj.17115)
Supplement: Supplementary file 2 — Table S1. Information of each plant sample and PSK treatment in RNA‐seq experiments. Table S2. W‐box motifs in the promoter regions of up‐ and downregulated DEGs. Table S3. W‐box motifs presence in the promoter regions of genes involved in PSK signaling. Table S4. Growth conditions and treatment methods for RNA‐seq data used in the comparison of PSK, PSY, flg22 and WCS417 induced DEGs. [file TPJ-120-2367-s008.zip › 3_sup-Tables.docx]

**Supplementary Table 1**

**
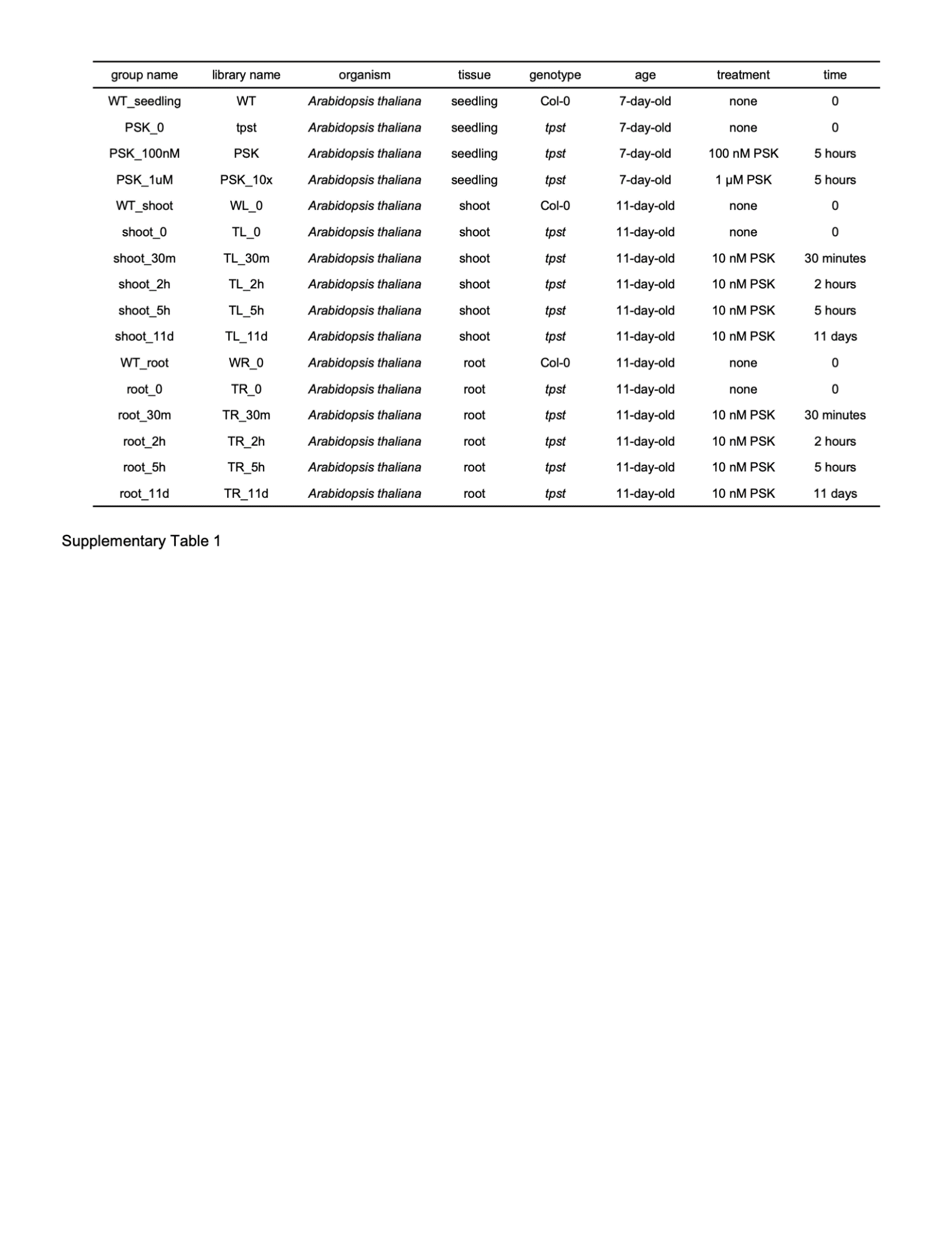
**

Supplementary Table 1. Information of each plant sample and PSK treatment in RNA-seq experiments. Group name of the plant sample was used in differential expression analysis, functional enrichment analysis and transcription factors enrichment analysis.

**Supplementary Table 2A**


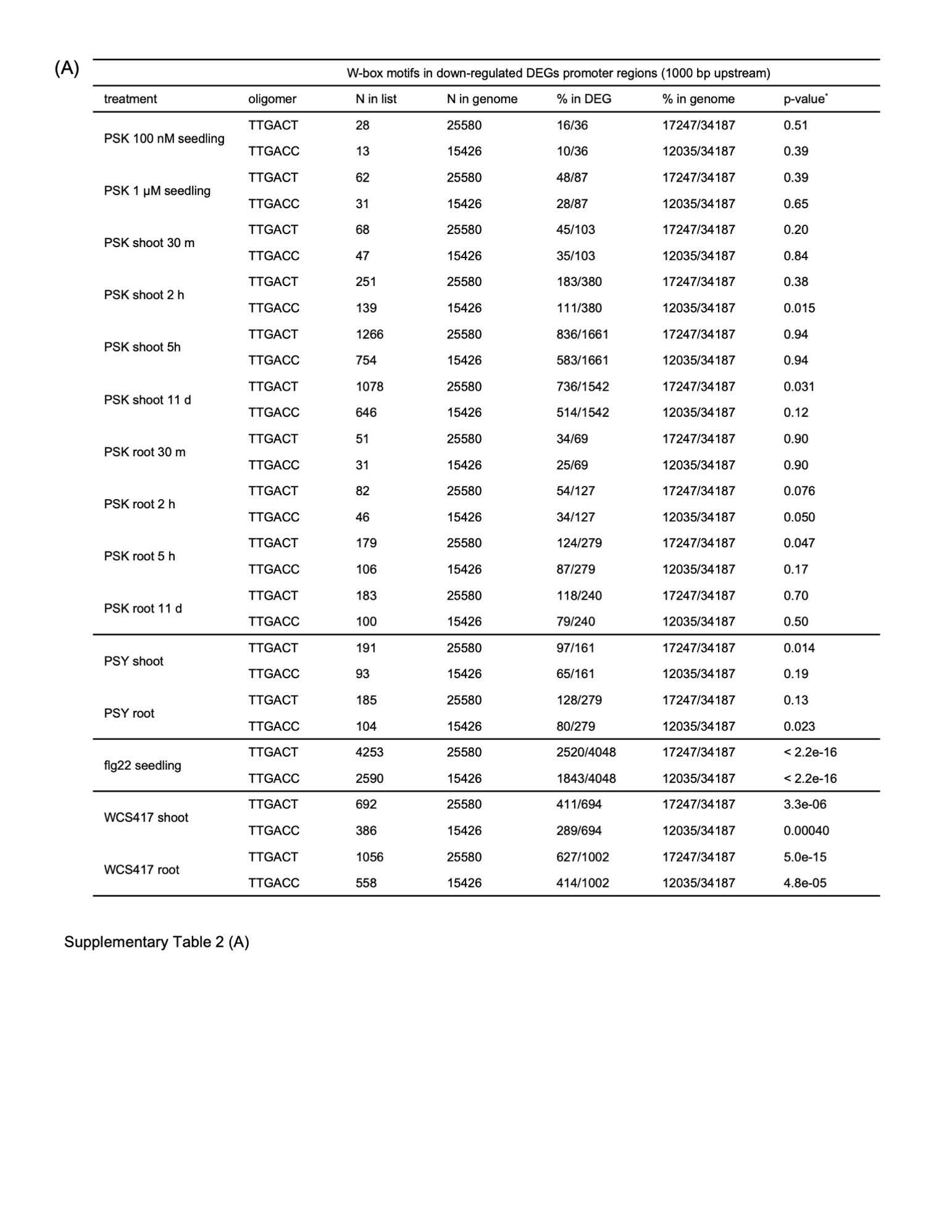


Supplementary Table 2. W-box motifs in the promoter regions of up- and downregulated DEGs. (A) Number of W-box motifs in the promoter regions (1000 bp upstream) of up-regulated DEGs, number of W-box motifs in the promoter regions of all genes in the genome, percentage of DEGs containing W-box motifs among all DEGs, and percentage of genes containing W-box motifs among all genes in each treatment condition.

^*^ The p-value represent Fisher’s exact test for the overrepresentation of DEGs containing W-box motifs compared to the genome background.

**Supplementary Table 2B**


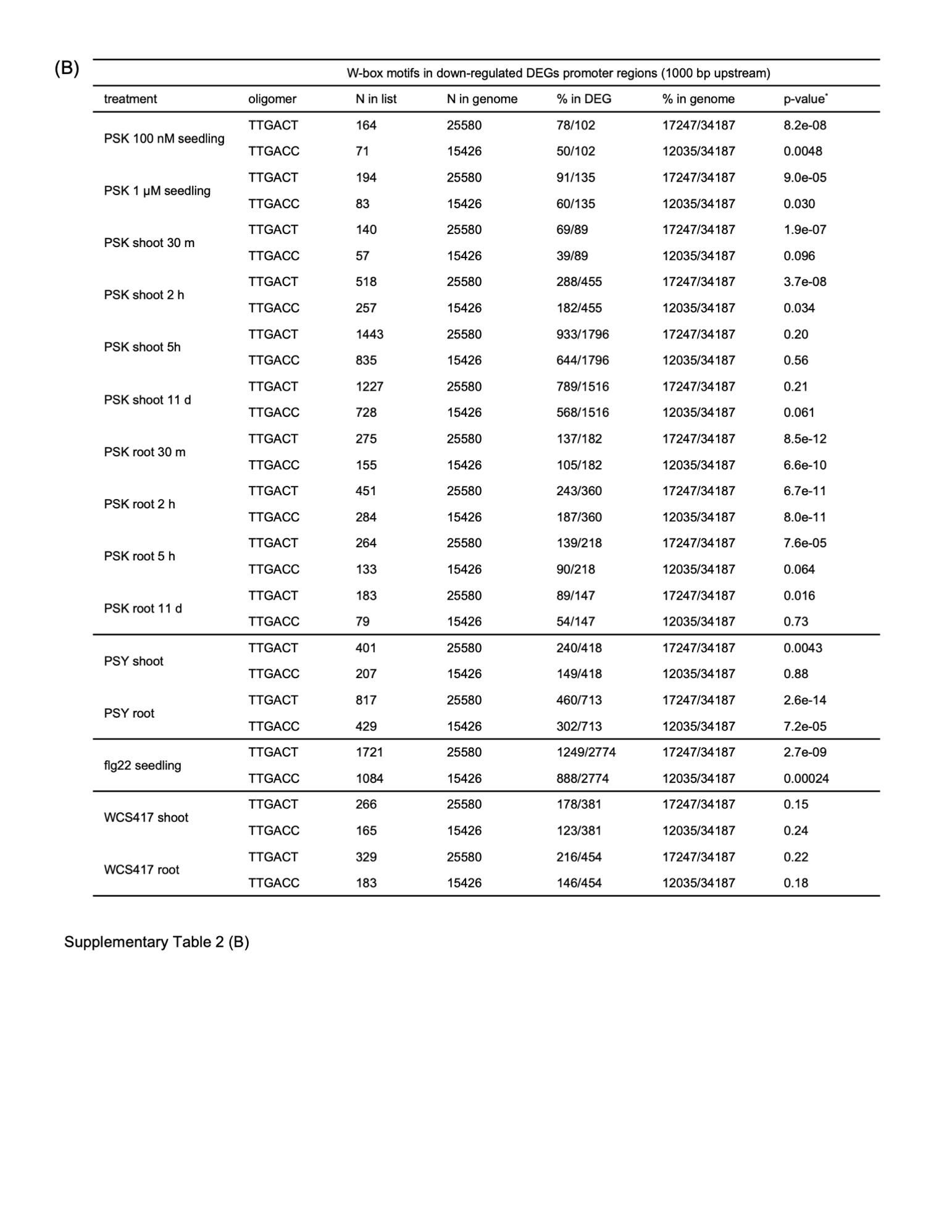


Supplementary Table 2. (B) Number of W-box motifs in the promoter regions (1000 bp upstream) of down-regulated DEGs, number of W-box motifs in the promoter regions of all genes in the genome, percentage of DEGs containing W-box motifs among all DEGs, and percentage of genes containing W-box motifs among all genes in each treatment condition.

^*^ The p-value represent Fisher’s exact test for the overrepresentation of DEGs containing W-box motifs compared to the genome background.

**Supplementary Table 3**


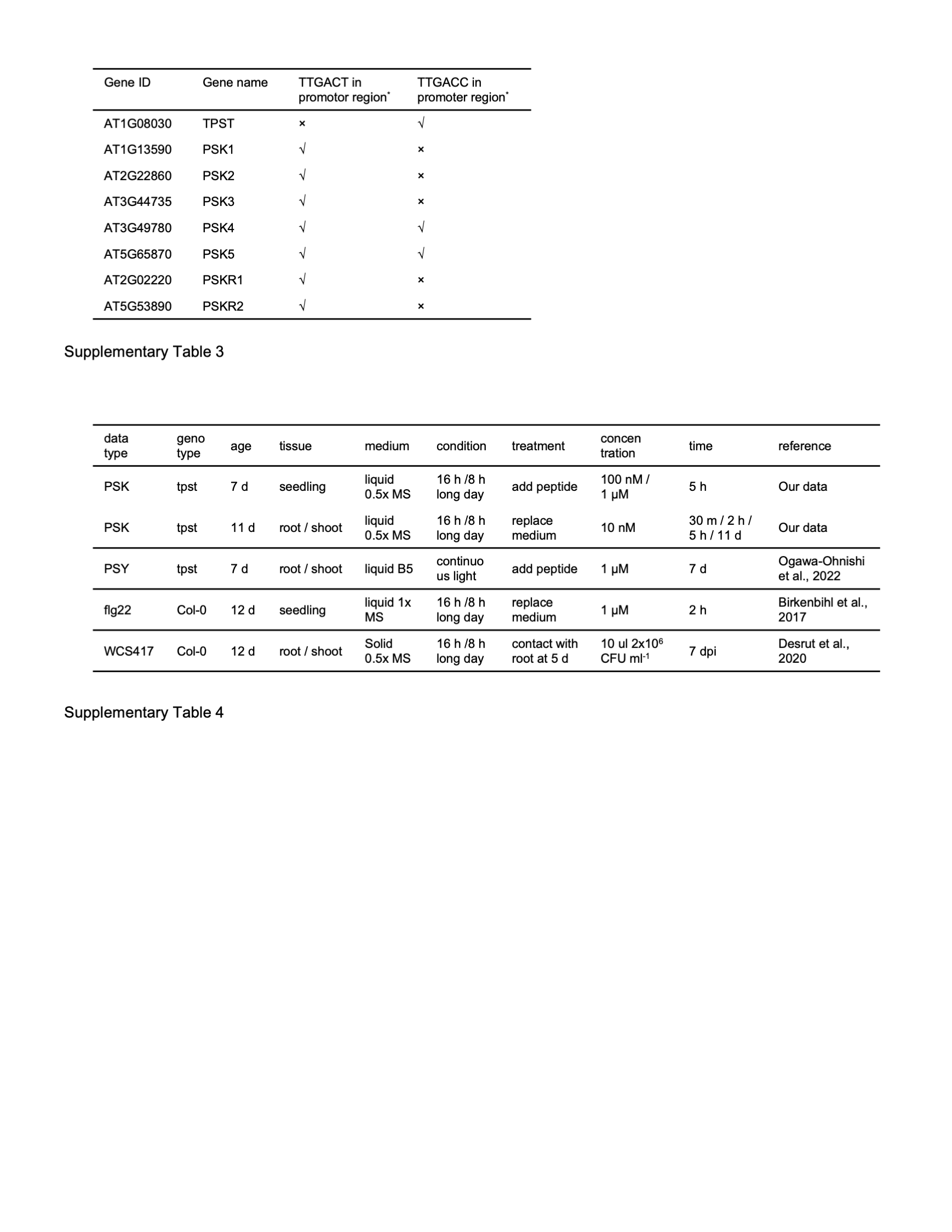


Supplementary Table 3. W-box motifs presence in the promoter regions of genes involved in PSK signaling.

^*^ √ indicates at least one W-box motif presents in promoter region; × indicates no W-box presents in promoter region.

**Supplementary Table 4**


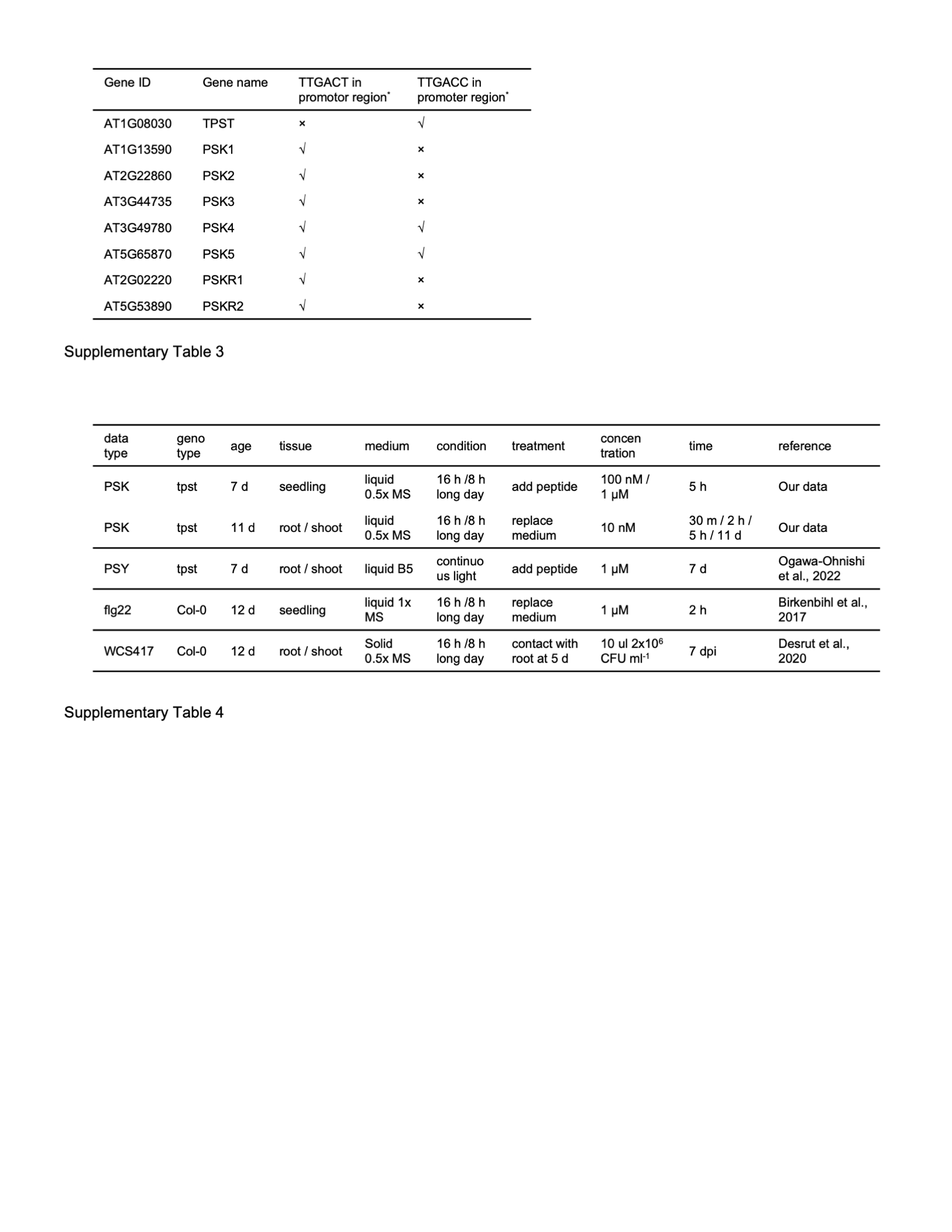


Supplementary Table 4. Growth conditions and treatment methods for RNA-seq data used in the comparison of PSK, PSY, flg22 and WCS417 induced DEGs.
