# Supplementary material for: Phytosulfokine downregulates defense‐related WRKY transcription factors and attenuates pathogen‐associated molecular pattern‐triggered immunity
Source: Plant J. 2024 Dec 11;120(6):2367–84. doi: 10.1111/tpj.17115 (PMC11658183; doi:10.1111/tpj.17115)
Supplement: Supplementary file 2 — Table S1. Information of each plant sample and PSK treatment in RNA‐seq experiments. Table S2. W‐box motifs in the promoter regions of up‐ and downregulated DEGs. Table S3. W‐box motifs presence in the promoter regions of genes involved in PSK signaling. Table S4. Growth conditions and treatment methods for RNA‐seq data used in the comparison of PSK, PSY, flg22 and WCS417 induced DEGs. [file TPJ-120-2367-s008.zip › 3_sup-Tables.pdf]

Supplementary Table 1

group name	library name	organism	tissue	genotype	age	treatment	time
WT_seedling	WT	*Arabidopsis thaliana*	seedling	Col-0	7-day-old	none	0
PSK_0	tpst	*Arabidopsis thaliana*	seedling	*tpst*	7-day-old	none	0
PSK_100nM	PSK	*Arabidopsis thaliana*	seedling	*tpst*	7-day-old	100 nM PSK	5 hours
PSK_1uM	PSK_10x	*Arabidopsis thaliana*	seedling	*tpst*	7-day-old	1 μM PSK	5 hours
WT_shoot	WL_0	*Arabidopsis thaliana*	shoot	Col-0	11-day-old	none	0
shoot_0	TL_0	*Arabidopsis thaliana*	shoot	*tpst*	11-day-old	none	0
shoot_30m	TL_30m	*Arabidopsis thaliana*	shoot	*tpst*	11-day-old	10 nM PSK	30 minutes
shoot_2h	TL_2h	*Arabidopsis thaliana*	shoot	*tpst*	11-day-old	10 nM PSK	2 hours
shoot_5h	TL_5h	*Arabidopsis thaliana*	shoot	*tpst*	11-day-old	10 nM PSK	5 hours
shoot_11d	TL_11d	*Arabidopsis thaliana*	shoot	*tpst*	11-day-old	10 nM PSK	11 days
WT_root	WR_0	*Arabidopsis thaliana*	root	Col-0	11-day-old	none	0
root_0	TR_0	*Arabidopsis thaliana*	root	*tpst*	11-day-old	none	0
root_30m	TR_30m	*Arabidopsis thaliana*	root	*tpst*	11-day-old	10 nM PSK	30 minutes
root_2h	TR_2h	*Arabidopsis thaliana*	root	*tpst*	11-day-old	10 nM PSK	2 hours
root_5h	TR_5h	*Arabidopsis thaliana*	root	*tpst*	11-day-old	10 nM PSK	5 hours
root_11d	TR_11d	*Arabidopsis thaliana*	root	*tpst*	11-day-old	10 nM PSK	11 days

Supplementary Table 1. Information of each plant sample and PSK treatment in RNA-seq experiments. Group name of the plant sample was used in differential expression analysis, functional enrichment analysis and transcription factors enrichment analysis.

Supplementary Table 2A

(A)

W-box motifs in down-regulated DEGs promoter regions (1000 bp upstream)						
treatment	oligomer	N in list	N in genome	% in DEG	% in genome	p-value*
PSK 100 nM seedling	TTGACT	28	25580	16/36	17247/34187	0.51
	TTGACC	13	15426	10/36	12035/34187	0.39
PSK 1 μM seedling	TTGACT	62	25580	48/87	17247/34187	0.39
	TTGACC	31	15426	28/87	12035/34187	0.65
PSK shoot 30 m	TTGACT	68	25580	45/103	17247/34187	0.20
	TTGACC	47	15426	35/103	12035/34187	0.84
PSK shoot 2 h	TTGACT	251	25580	183/380	17247/34187	0.38
	TTGACC	139	15426	111/380	12035/34187	0.015
PSK shoot 5h	TTGACT	1266	25580	836/1661	17247/34187	0.94
	TTGACC	754	15426	583/1661	12035/34187	0.94
PSK shoot 11 d	TTGACT	1078	25580	736/1542	17247/34187	0.031
	TTGACC	646	15426	514/1542	12035/34187	0.12
PSK root 30 m	TTGACT	51	25580	34/69	17247/34187	0.90
	TTGACC	31	15426	25/69	12035/34187	0.90
PSK root 2 h	TTGACT	82	25580	54/127	17247/34187	0.076
	TTGACC	46	15426	34/127	12035/34187	0.050
PSK root 5 h	TTGACT	179	25580	124/279	17247/34187	0.047
	TTGACC	106	15426	87/279	12035/34187	0.17
PSK root 11 d	TTGACT	183	25580	118/240	17247/34187	0.70
	TTGACC	100	15426	79/240	12035/34187	0.50
PSY shoot	TTGACT	191	25580	97/161	17247/34187	0.014
	TTGACC	93	15426	65/161	12035/34187	0.19
PSY root	TTGACT	185	25580	128/279	17247/34187	0.13
	TTGACC	104	15426	80/279	12035/34187	0.023
flg22 seedling	TTGACT	4253	25580	2520/4048	17247/34187	< 2.2e-16
	TTGACC	2590	15426	1843/4048	12035/34187	< 2.2e-16
WCS417 shoot	TTGACT	692	25580	411/694	17247/34187	3.3e-06
	TTGACC	386	15426	289/694	12035/34187	0.00040
WCS417 root	TTGACT	1056	25580	627/1002	17247/34187	5.0e-15
	TTGACC	558	15426	414/1002	12035/34187	4.8e-05

Supplementary Table 2. W-box motifs in the promoter regions of up- and downregulated DEGs. (A) Number of W-box motifs in the promoter regions (1000 bp upstream) of up-regulated DEGs, number of W-box motifs in the promoter regions of all genes in the genome, percentage of DEGs containing W-box motifs among all DEGs, and percentage of genes containing W-box motifs among all genes in each treatment condition.

* The p-value represent Fisher's exact test for the overrepresentation of DEGs containing W-box motifs compared to the genome background.

Supplementary Table 2B

(B)

W-box motifs in down-regulated DEGs promoter regions (1000 bp upstream)						
treatment	oligomer	N in list	N in genome	% in DEG	% in genome	p-value*
PSK 100 nM seedling	TTGACT	164	25580	78/102	17247/34187	8.2e-08
	TTGACC	71	15426	50/102	12035/34187	0.0048
PSK 1 μM seedling	TTGACT	194	25580	91/135	17247/34187	9.0e-05
	TTGACC	83	15426	60/135	12035/34187	0.030
PSK shoot 30 m	TTGACT	140	25580	69/89	17247/34187	1.9e-07
	TTGACC	57	15426	39/89	12035/34187	0.096
PSK shoot 2 h	TTGACT	518	25580	288/455	17247/34187	3.7e-08
	TTGACC	257	15426	182/455	12035/34187	0.034
PSK shoot 5h	TTGACT	1443	25580	933/1796	17247/34187	0.20
	TTGACC	835	15426	644/1796	12035/34187	0.56
PSK shoot 11 d	TTGACT	1227	25580	789/1516	17247/34187	0.21
	TTGACC	728	15426	568/1516	12035/34187	0.061
PSK root 30 m	TTGACT	275	25580	137/182	17247/34187	8.5e-12
	TTGACC	155	15426	105/182	12035/34187	6.6e-10
PSK root 2 h	TTGACT	451	25580	243/360	17247/34187	6.7e-11
	TTGACC	284	15426	187/360	12035/34187	8.0e-11
PSK root 5 h	TTGACT	264	25580	139/218	17247/34187	7.6e-05
	TTGACC	133	15426	90/218	12035/34187	0.064
PSK root 11 d	TTGACT	183	25580	89/147	17247/34187	0.016
	TTGACC	79	15426	54/147	12035/34187	0.73
PSY shoot	TTGACT	401	25580	240/418	17247/34187	0.0043
	TTGACC	207	15426	149/418	12035/34187	0.88
PSY root	TTGACT	817	25580	460/713	17247/34187	2.6e-14
	TTGACC	429	15426	302/713	12035/34187	7.2e-05
flg22 seedling	TTGACT	1721	25580	1249/2774	17247/34187	2.7e-09
	TTGACC	1084	15426	888/2774	12035/34187	0.00024
WCS417 shoot	TTGACT	266	25580	178/381	17247/34187	0.15
	TTGACC	165	15426	123/381	12035/34187	0.24
WCS417 root	TTGACT	329	25580	216/454	17247/34187	0.22
	TTGACC	183	15426	146/454	12035/34187	0.18

Supplementary Table 2. (B) Number of W-box motifs in the promoter regions (1000 bp upstream) of down-regulated DEGs, number of W-box motifs in the promoter regions of all genes in the genome, percentage of DEGs containing W-box motifs among all DEGs, and percentage of genes containing W-box motifs among all genes in each treatment condition.

* The p-value represent Fisher's exact test for the overrepresentation of DEGs containing W-box motifs compared to the genome background.

Supplementary Table 3

Gene ID	Gene name	TTGACT in promotor region*	TTGACC in promoter region*
AT1G08030	TPST	×	√
AT1G13590	PSK1	√	×
AT2G22860	PSK2	√	×
AT3G44735	PSK3	√	×
AT3G49780	PSK4	√	√
AT5G65870	PSK5	√	√
AT2G02220	PSKR1	√	×
AT5G53890	PSKR2	√	×

Supplementary Table 3. W-box motifs presence in the promoter regions of genes involved in PSK signaling.

* √ indicates at least one W-box motif presents in promoter region; × indicates no W-box presents in promoter region.

Supplementary Table 4

data type	geno type	age	tissue	medium	condition	treatment	concen tration	time	reference
PSK	tpst	7 d	seedling	liquid 0.5x MS	16 h /8 h long day	add peptide	100 nM / 1 µM	5 h	Our data
PSK	tpst	11 d	root / shoot	liquid 0.5x MS	16 h /8 h long day	replace medium	10 nM	30 m / 2 h / 5 h / 11 d	Our data
PSY	tpst	7 d	root / shoot	liquid B5	continuo us light	add peptide	1 µM	7 d	Ogawa-Ohnishi et al., 2022
flg22	Col-0	12 d	seedling	liquid 1x MS	16 h /8 h long day	replace medium	1 µM	2 h	Birkenbihl et al., 2017
WCS417	Col-0	12 d	root / shoot	Solid 0.5x MS	16 h /8 h long day	contact with root at 5 d	10 ul $2x10^{6}$ CFU ml^{-1}	7 dpi	Desrut et al., 2020

Supplementary Table 4. Growth conditions and treatment methods for RNA-seq data used in the comparison of PSK, PSY, flg22 and WCS417 induced DEGs.
